
JOURNAL INFORMATION
==============================
NLM Title Abbreviation: Insights Imaging
Journal ID: Insights Imaging
NLM Journal ID: 101532453

Insights into Imaging

EISSN: 1869-4101
Publisher: Springer

ARTICLE INFORMATION
==============================
PMCID: PMC6773793
PMID: 31576431
DOI: 10.1186/s13244-019-0779-6
Article ID: 779
Article version: 1
Subjects: Meeting Abstracts

ESHNR 2019 Book of Abstracts

Palermo, Italy.

Electronic publication date: 2019 Oct 2
Publication date: 2019 Oct
Volume: 10
Issue: Suppl 3
Issue sponsor: Publication costs were not supported by sponsorship.
Electronic Location ID: 88
Copyright: © The Author(s). 2019
License: Open AccessThis article is distributed under the terms of the Creative Commons Attribution 4.0 International License (http://creativecommons.org/licenses/by/4.0/), which permits unrestricted use, distribution, and reproduction in any medium, provided you give appropriate credit to the original author(s) and the source, provide a link to the Creative Commons license, and indicate if changes were made.
License URL: https://creativecommons.org/licenses/by/4.0/

Conference name: 32nd Annual Meeting and Refresher Course of the European Society of Head and Neck Radiology
Conference Acronym: ESHNR 2019
Conference location: Palermo, Italy
Conference date: 3 - 5 October 2019
issue-copyright-statement: © The Author(s) 2019

==============================
eshnr
european society of
head and neck radiology

### Insights into Imaging

Education and strategies in European radiology

ESHNR 2019 Book of Abstracts / Volume 10 / Supplement 3 / October 2019

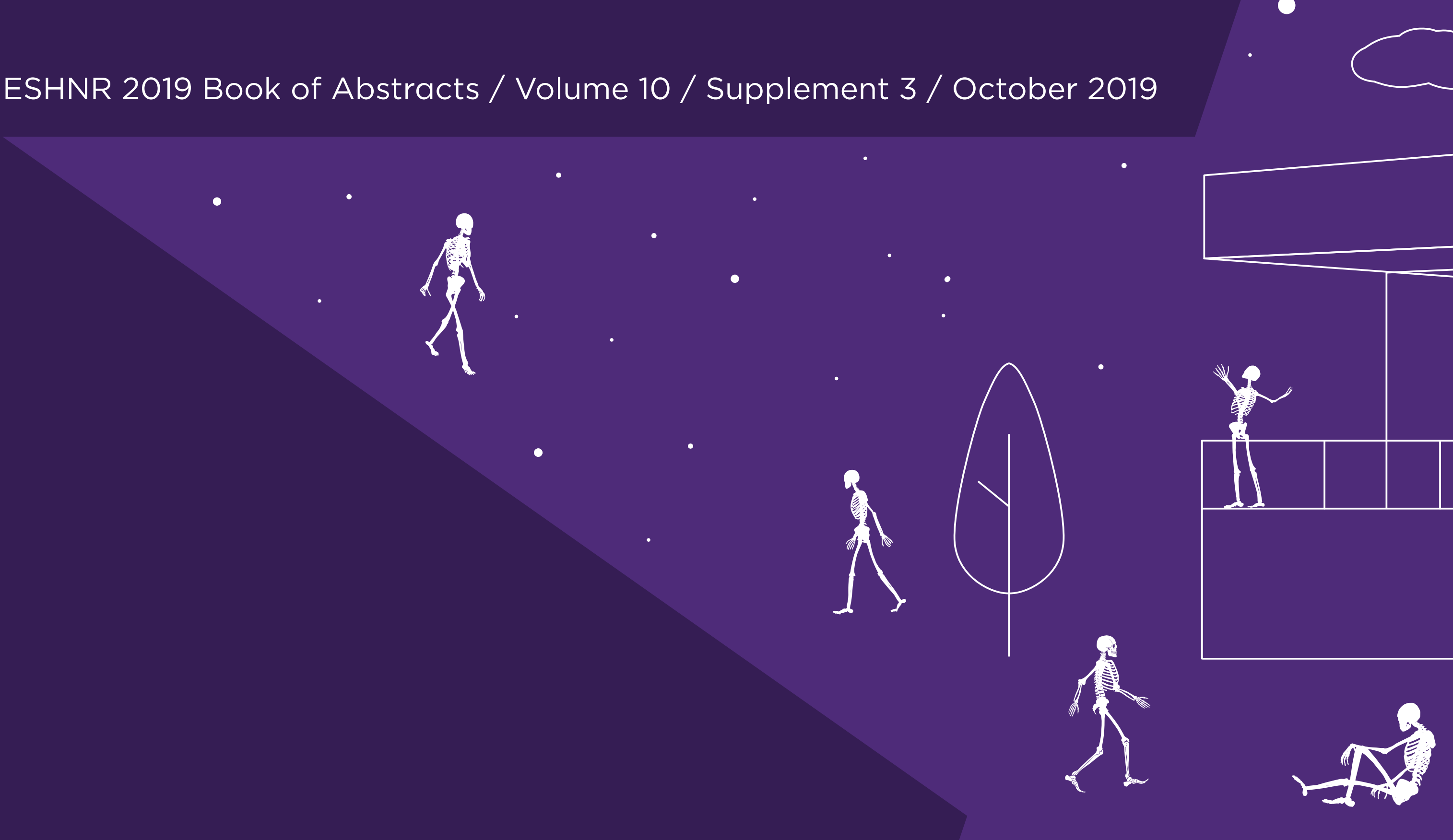

ESHNR 2019, 32nd Annual Meeting and Refresher Course
October 3–5, 2019, Cinisi, Palermo/IT

ESR
EUROPEAN SOCIETY
OF RADIOLOGY

eshnr european society of
head and neck radiology

### ESHNR 2019

32nd Annual Meeting and Refresher Course

**October 3–5, 2019**
**Cinisi, Palermo/IT**

www.eshnr.eu

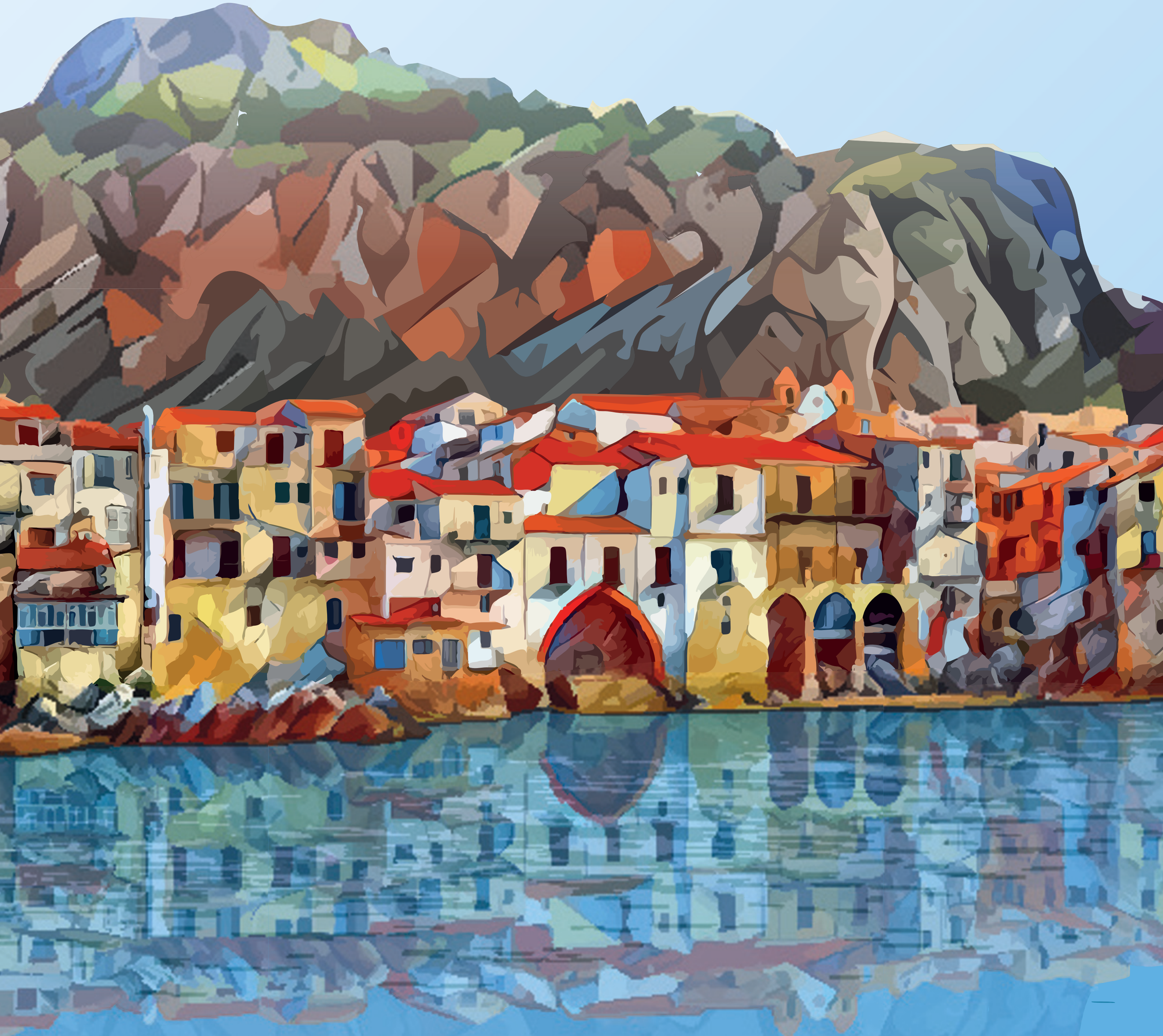

### IMPORTANT ADDRESSES

### Organising Secretariat

ESHNR Office
Am Gestade 1
1010 Vienna/Austria
Phone: +43 1 5334064-918
E-Mail: office@eshnr.eu
www.eshnr.eu

### Conference Venue

Florio Park Hotel
Via Impastato Peppino 41
90045 Cinisi PA/Italy

### CME CREDITS

The “ESHNR European Society of Head and Neck Radiology” (or) “ESHNR 2019 – $32^{nd}$ ANNUAL MEETING AND REFRESHER COURSE” is accredited by the European Accreditation Council for Continuing Medical Education (EACCME) to provide the following CME activity for medical specialists. The EACCME is an institution of the European Union of Medical Specialists (UEMS), www.uems.net

The $32^{nd}$ Annual Meeting of the European Society of Head and Radiology, Palermo, Italy, 03/10/2019-05/10/2019 has been accredited by the European Accreditation Council for Continuing Medical Education (EACCME®) with 17 European CME credits (ECMEC®s). Each medical specialist should claim only those hours of credit that he/she actually spent in the educational activity

Insights Imaging (2019) 10 (Suppl. 3): 88
https://doi.org/10.1186/s13244-019-0779-6
Published Online: 02 October 2019

### ESHNR EXECUTIVE COMMITTEE

### President
Agnieszka Trojanowska, Lublin/PL

### Vice President
Bert De Foer, Antwerp/BE

### Past President
Christian Czerny, Vienna/AT

### Secretary
Davide Farina, Brescia/IT

### Treasurer
Heidi Beate Eggesbø, Oslo/NO

### Fellows
Timothy Beale, London/UK
Minerva Becker, Geneva/CH
Sotirios Bisdas, London/UK
Romain Kohler, Sion/CH
Soraya Robinson, Vienna/AT
Arthur Varoquaux, Marseille/FR
Berit Verbist, Leiden/NL

### Fellow At Large
Salman Qureshi, Manchester/UK

### Meeting President 2019
Antonio Lo Casto, Palermo/IT

### Future Meeting Presidents
Christian Czerny, Vienna/AT
Soraya Robinson, Vienna/AT

### ESHNR 2019 SCIENTIFIC COMMITTEE

Minerva Becker, Geneva/CH
Sotirios Bisdas, London/UK
Christian Czerny, Vienna/AT
Heidi Eggesbø, Oslo/NO
Romain Kohler, Sion/CH
Antonio Lo Casto, Palermo/IT
Salman Qureshi, Manchester/UK
Soraya Robinson, Vienna/AT
Agnieszka Trojanowska, Lublin/PL

### TABLE OF CONTENTS

Scientific Oral Presentation Session, Thursday, October 3 (SOPS 1&2) 7–25
Scientific Oral Presentation Session, Friday, October 4 (SOPS 3) 26–31
Authors' Index 32–33

eshnr european society of
head and neck radiology

### ESHNR 2019 SCIENTIFIC SESSIONS

### SCIENTIFIC ORAL PRESENTATION SESSION: ONCOLOGY/SALIVARY

### SOPS 1.1

### Correlation between Histogram-based parameters of DCE-MRI and metabolic 18F-FDG PET parameters in OPSCC: Evaluation in primary tumors and metastatic nodes

*E. Gangemi, A. Vidiri, E. Ruberto, R. Pasqualoni, A. Farneti, R. Pellini, G. Sanguineti, S. Marzi; Rome/Italy*

Short Summary: Different imaging modalities have been used for diagnosis and treatment monitoring in oropharyngeal squamous cell carcinoma (OPSCC). Advanced MRI techniques, as DWI and Dynamic Contrast-Enhanced MRI (DCE-MRI), and PET imaging have been proposed for a comprehensive tumor characterization, including tissue architecture, microvascularity and metabolic activity. Although it is known that tumoral blood flow and vessel permeability may affect the FDG uptake, only a few studies have investigated the relationships among 18F-FDG PET and DCE-MRI parameters, reporting controversial results.

Purpose/Objectives: To investigate the correlation between histogram-based DCE-MRI parameters and 18F-FDG-PET values in OPSCC, both in primary tumor (PT) and in metastatic nodes (LNs).

Methods and Materials: 52 patients with a new diagnosis of OPSCC were enrolled in the present study. Imaging including DCE-MRI and F-18 FDG PET/CT was acquired in all patients. Both PT and the largest LN, if present, were volumetrically contoured. Semi-quantitative and quantitative parameters, including the transfer constant $K^{trans}$, were calculated from DCE-MRI. The percentiles, P10, P25, P50, P75,P90, and skewness, kurtosis and entropy were obtained from the histogram-based analysis of each perfusion parameter. Standardized uptake values, SUVmax, SUVpeak, SUVmean, metabolic tumor volume MTV and total lesion glycolysis TLG were calculated using a SUV threshold of 40%, on a dedicated workstation. The correlations between all variables were investigated with the Spearman-rank correlation test. To exclude false positive results under multiple testing, Benjamini and Hockberg method was applied.

Results: No significant correlation was found in PT, while significant associations emerged in LN between $K^{trans}$ and some PET parameters, as shown in Figure 1.

*Figure 1: Spearman-rank correlation test between $K^{trans}$ and PET parameters in LN*

| | | SUVMAX | SUVPEAK | SUVMEAN | TLG | MTV |
|---|---|---|---|---|---|---|
| **ktransP10** | correlation coefficient | **-0,375** | -,330’ | **-,369’** | -,098 | ,001 |
| | p | **,011** | ,027 | **,013** | ,524 | ,993 |
| **ktransP25** | correlation coefficient | **-,384’’** | -346’ | **-,378’** | -,168 | -,081 |
| | p | **,009** | ,020 | **,011** | ,271 | ,599 |
| **ktransP50** | correlation coefficient | **-,405’’** | **-,372’** | **-,394’’** | -,226 | -,126 |
| | p | **,006** | **,012** | **,007** | ,135 | ,408 |
| **ktransP75** | correlation coefficient | **-,429’’** | **-,424’’** | **-,421’’** | -,348’ | -,236 |
| | p | **,003** | **,004** | **,004** | ,019 | ,119 |
| **ktransP90** | correlation coefficient | **-,433’’** | **-,433’’** | **-,438** | **-,402** | -,270 |
| | p | **,003** | **,002** | **,003** | **,006** | ,073 |
| **ktransSKEWNESS** | correlation coefficient | ,269 | ,221 | ,234 | ,123 | ,054 |
| | p | ,075 | ,145 | ,122 | ,419 | ,724 |
| **ktransKURTOSIS** | correlation coefficient | ,285 | ,254 | ,260 | ,199 | ,110 |
| | p | ,057 | ,092 | ,085 | ,190 | ,472 |
| **ktransENTROPY** | correlation coefficient | -,296’ | -,299’ | -,279 | -,273 | -,223 |
| | p | ,048 | ,046 | ,063 | ,070 | ,141 |

**significant correlations are in bold, corresponding p values are red.*

Conclusion: The evident relationships between DCE-MRI parameters and 18F-FDG-PET in OPSCC imply a complex interaction between microvascular properties and tumour metabolism. The reason why these associations were found only for LNs is not clear. We hypothesize that this may be due to the complexity of the primary tumor, where a lot of factors such as inflammation/ulceration influence the tumoral microenvironment, probably intefering and making these relationships less clear than in lymph-nodes. DCE-MRI and 18F-FDG-PET provide complementary information but a better understanding of the underlying mechanisms is advisable.

Disclosure: No significant relationships.
Keywords: 18F-FDG-PET, Histogram analysis, OPSCC, DCE-MRI

### SOPS 1.2

### Interobserver agreement of ADC value measurements in stage 3-4 H&N tumours pre and post-chemoradiotherapy and reliability compared to interval post treatment ADC changes

*M. Anjari, A. Guha, C. Burd, M. Varela, M. Lei, V. Goh, S. Connor; London/United Kingdom*

Short Summary: Stage 3-4 head and neck (H&N) squamous cell carcinoma at specific sites is primarily treated by chemoradiotherapy. Diffusion-weighted MRI (DWI) may allow earlier detection and intervention for residual/recurrent tumour but measurements must be reliable. Interobserver variations in apparent diffusion coefficient (ADC) measurements performed pre- and post-treatment has not been evaluated.

Purpose/Objectives: To measure interobserver agreement in ADC values at disease sites measured at baseline, 6 and 12 weeks post chemoradiotherapy in patients with stage 3-4 H&N cancer, and assess reliability compared with interval changes in the ADC values.

Methods and Materials: 25 adult patients (21 male, mean age 59.5 years) in whom curative chemo-radiotherapy was planned and there was >1 cm of measurable disease at primary or nodal site were included. They underwent MRI including DWI sequences and calculation of ADC maps at baseline and 6 and 12 weeks post chemoradiotherapy. Two independent radiologists delineated regions of interest (ROIs) and measured ADC values at primary tumour sites and the largest pathological lymph node according to areas demonstrating DWI hyperintensity. Three different methods were used to define the ROI: a volumetric ROI around the whole of the tumour/node, an ROI outlining a representative slice of the tumour/node, and a sample of the representative slice excluding any areas of necrosis. If there was no longer a focus of restricted diffusion, a standardised 6mm diameter ROI was placed at its original location (termed non-measurable).

Results: There was excellent interobserver agreement in mean ADC measurement within volumetric ROIs separately delineating the whole of the tumour and the largest involved node at baseline and on a slice within the largest disease burden, assessed using the Bland-Altman method and paired t-tests (Table 1). Agreement remained good at ROIs placed only on a single slice excluding areas of necrosis, and at ROIs placed on the original tumour and nodal site on post-treatment images. There was a marked rise in ADC at both node and primary tumour site between baseline and 12 weeks (Table 2) that was significantly greater than interobserver differences in measured ADC at both timepoints.

**Table 1:** *Interobserver comparison of ADC measurements at baseline and post-treatment*

| | Baseline ADC Measurements | | | | | | 6 weeks post-treatment ADC Measurements | | | | | | 12 weeks post-treatment ADC Measurement | | | | | |
|---|---|---|---|---|---|---|---|---|---|---|---|---|---|---|---|---|---|---|
| | Node volume | Node representative slice | Node slice sample | Tamour volume | Tumour representative slice | Tomour slice sample | Node volume | Node representative slice | Node slice sample/NM disease ROI | Tumour volume | Tumour representative slice | Tumour slice sample/NM disease ROI | Node volume | Node representative slice | Node slice sample/NM disease ROI | Tumour volume | Tumour representative slice | Tumour slice sample/NM disease ROI |
| #Observations | 24 | 24 | 24 | 22 | 22 | 22 | 14 | 14 | 24 | 2 | 2 | 22 | 5 | 5 | 23 | 0 | 0 | 21 |
| Estimated Bias | 8,59 | -15,45 | 14,52 | -8,68 | -26,30 | 7,65 | -30,08 | -14,54 | 6,53 | -112,33 | -380,18 | -42,02 | -60,65 | -56,12 | 7,48 | - | - | -10,88 |
| Limits of Agreement | 119,38 | 108,43 | 136,68 | 122,65 | 101,47 | 90,84 | 136,13 | 68,17 | 99,96 | 227,64 | 174,33 | 289,97 | 158,48 | 178,32 | 249,87 | - | - | 233,11 |
| Paired t-test p Value | 0,50 | 0.18 | 0,32 | 0,52 | 0,27 | 0,45 | 0,13 | 0,14 | 0,54 | 0,40 | 0,10 | 0,20 | 0,17 | 0,24 | 0,78 | - | - | 0,68 |
| Lower Confidence Interval | -17,12 | -38,81 | -14,93 | -36,43 | -49,25 | -12,90 | -70,18 | -34,62 | -15,01 | -1155,82 | -1179,32 | -107,62 | -161,05 | 169,08 | -47,65 | - | - | -65,02 |
| Upper Confidence Interval | 34,31 | 7,91 | 43,96 | 19,07 | 3,35 | 28,19 | 10,02 | 5,55 | 28,07 | 931,16 | 418,95 | 23,57 | 39,75 | 56,85 | 62,61 | - | - | 43,26 |
| ICC | 0,98 | 0,98 | 0,94 | 0,97 | 0,98 | 0,98 | 0,95 | 0,98 | 0,97 | 0,45 | 0,33 | 0,86 | 0,91 | 0,93 | 0,86 | - | - | 0,94 |

There was no statistically significant interobserver difference in ADC values from paired ROIs placed within involved nodes or primary tumour either at baseline or following chemoradiotherapy. At each timepoint, this was true whether ADC was measured from volumetric ROI's including the whole of the node/ tumour, a 2D ROI from a delected slice within these volumes or just including part oft he slice containing disease and excluding necrotic tissue. Following treatment, if a node/tumour was no longer measurable. ADC values were recorded from 6mm diameter ROI at the site of previously seen disease.

**Table 2:** *Change in ADC measurements between baseline and 12 weeks post-treatment*

| | Node volume | Node representative slice | Node slice sample/NM disease ROI | Tumour Volume | Tumour representative slice | Tumour slice sample/NM disease ROI |
|---|---|---|---|---|---|---|
| Limits of Agreement | 669.97 | 716.42 | 633.91 | 700.45 | 699.16 | 689.41 |
| Paired t-test p Value | 0.04 | 0.03 | <0.001 | <0.001 | <0.001 | <0.001 |
| Lower Confidence Interval | -876.92 | -1016.03 | -743.87 | -946.27 | -996.24 | -1062.32 |
| Upper Confidence Interval | -28.07 | -108.32 | -464.15 | -621.92 | -671.49 | -742.10 |
| ICC | <0.001 | <0.001 | <0.001 | 0.05 | 0.06 | 0.05 |

Conclusion: Even in the presence of significant post-treatment change, ADC values at existing or previous sites of H&N cancer can be reliably obtained and so may be suitable as an imaging biomarker to assess treatment response earlier than conventional methods allow.

Disclosure: No significant relationships.
Keywords: Cancer, DWI, Reliability, Treatment, ADC

### SOPS 1.3

### T1rho MRI of nasopharyngeal carcinoma and benign hyperplasia in the nasopharynx

*Q. Ai, W. Chen, W.J. Lam, D.M. Poon, A.D. King; Shatin, N.T./Hong Kong*

Short Summary: Anatomical MRI can detect nasopharyngeal carcinoma (NPC), but benign hyperplasia in the nasopharynx decreases the accuracy for anatomical MRI. T1rho imaging is a functional technique that investigate the micro-environment in tissue and so may be able to use to distinguish these two disease. Our study found that t1rho imaging was able to differentiate NPC from benign hyperplasia and thus may have the potential to be used in the detection of NPC.

Purpose/Objectives: The importance of anatomical MRI in the detection of nasopharyngeal carcinoma (NPC) has been recently established. However, benign hyperplasia (BH) in the nasopharynx may decrease the accuracy of MRI in the detection of NPC. T1rho imaging can detect the chemical exchange between water associated with macromolecules and free water in the tissues, and so has potentials to distinguish NPC from BH. This study aimed to evaluate the ability of t1rho imaging for distinguishing NPC from BH in the nasopharynx.

Methods and Materials: Thirty-one patients with biopsy-confirmed NPC and 28 patients with benign hyperplasia prospectively underwent t1rho imaging using a frequency of spin-lock of 400 Hz and 5 spin-lock time (0, 10, 30, 55 and 90 ms). Mean, SD, skewness and kurtosis of t1rho values were calculated. T1rho parameters were compared between the two groups using the Mann-Whitney U-test. Receiver-operating characteristics analysis with the area under the curve (AUC) was used to identify the optimal threshold and the diagnostic performance was calculated. A p-value of $< 0.05$ was considered statistically significant.

Results: Compared to benign hyperplasia, NPC showed a significantly lower t1rho mean ($68.1 \pm 5.9$ vs. $77.3 \pm 8.0$ ms, $p < 0.001$) and SD ($32.1 \pm 32.4$ vs. $61.0 \pm 42.8$ ms, $p = 0.005$). There was no significant difference in skewness ($p = 0.313$) or kurtosis ($p = 0.963$) between the two groups. The AUCs of the t1rho mean and SD were 0.831 and 0.813, respectively (all $p < 0.001$). An optimal threshold of a t1rho mean of < 70.0 ms for identifying early NPC showed a sensitivity of 74.2%, specificity of 85.7%, positive predictive value of 85.2%, negative predictive value of 75.0% and an accuracy of 79.7%.

Conclusion: T1rho imaging is a new functional MRI technique that has potential to differentiate NPC from benign hyperplasia and the mean value was a promising parameter to be used in the detection of NPC.

Disclosure: No significant relationships.
Keywords: T1rho imaging, MRI, nasopharyngeal carcinoma, benign hyperplasia, head and neck tumour

### SOPS 1.4

### CT and MRI findings of glomangiopericytoma in the head and neck: case series study and systemic review

*H.J. Kim, C.H. Suh, J.H. Lee, L.H. Joo, M.K. Lee, S.J. Cho; Seoul/Korea, Republic of*

Short Summary: Glomangiopericytoma is a rare sinonasal mesenchymal tumor categorized as being of borderline or low malignant potential. We reviewed the CT and MRI findings of glomangiopericytoma in the head and neck, including those on diffusion-weighted image(DWI) and dynamic contrast-enhanced(DCE) MRI, via a retrospective case series study and systematic review. Our study revealed that glomangiopericytoma is a well-defined lobulated strongly-enhancing soft tissue mass with erosive bony remodeling. Typically, it is hyperintense on T2WI with vascular signal voids, has a high mean ADC value, and a wash-in and wash-out pattern on DCE MRI. Although the CT findings are non-specific, typical MRI findings including those on the ADC map and DCE MRI could be helpful for differentiating glomangiopericytomas from other hypervascular tumors in the head and neck.

Purpose/Objectives: The purpose is to review the CT and MRI findings of glomangiopericytoma in the head and neck, including the findings on DWI and DCE MRI, via a retrospective study of case series and a systemic review.

Methods and Materials: Patients with pathologically confirmed glomangiopericytoma who underwent preoperative CT or MRI were electronically searched and included in this study. Two neuroradiologists independently reviewed CT and MR imaging findings including DWI and DCE MRI. For a systematic review, a systemic search of PubMed and Embase was performed using the following search terms: ((glomangiopericytoma)) AND ((“computed tomography”) OR (CT) OR (“magnetic resonance imaging”) OR (“MR imaging”) OR (MRI)).

Results: Six patients (M:F=3:3; range, 48-79 years) satisfied the inclusion criteria. All had CT examinations, and four of them underwent MRI. Glomangiopericytomas were well-defined soft tissue masses with frequent erosive bone remodeling on CT. On MRI, they showed intermediate to high signal intensity, and all of them had vascular signal voids on T2WI. Three glomangiopericytomas in whom DWI was available demonstrated high mean ADC values (range, $1.27 - 2.09 \times 10^{-3}$ $mm^2/s$). On DCE MRI (n=2), glomangiopericytomas revealed a rapid wash-in and wash-out pattern. The systematic search yielded nine case reports after removal of duplicate articles and those which were not in the field of interest. They showed similar CT and MRI findings to ours. None of the articles reported DWI or DCE MRI findings.

Conclusion: Glomangiopericytoma is a well-defined strongly-enhancing soft tissue mass with erosive bony remodeling. It shows hyperintense signal on T2WI with vascular signal voids, a high mean ADC value, and a wash-in and wash-out pattern on DCE MRI, which can be helpful for differentiating it from other hypervascular tumors, especially in the sinonasal cavity.

Disclosure: No significant relationships.
Keywords: ADC, Systematic review, glomangiopericytoma, MRI, CT, DCE

### SOPS 1.5

**Diffusion weighted and dynamic contrast-enhanced MRI as a predictor of treatment response to chemoradiotherapy in head and neck cancer**

*M. Garbajs[1], U. Zabret[1], S. Bisdas[2], P. Strojan[1], K. Šurlan Popovič[1]; [1]Ljubljana/Slovenia, [2]London/United Kingdom*

Short Summary: In locoregionally advanced Head and Neck Squamous Cell Carcinoma (HNSCC), intra-treatment scanning early during the course of Concomitant Chemoradiotherapy (CRT) with advanced functional Magnetic Resonance Imaging (MRI) could potentially allow for modification of treatment to maximize the chance of favorable outcome.

Purpose/Objectives: The aim of this prospective study was to assess the predictive value of parameters derived from Dynamic Contrast-Enhanced (DCE) and Diffusion Weighted (DW) MRI for treatment response early during the course of CRT in patients with HNSCC.

Methods and Materials: MRI scans were performed in 20 patients with locoregionally advanced HNSCC at baseline, after 10 Grays (Gy) and 40 Gy of CRT. DW and DCE derived parameters as well as the volumes were measured from primary tumours and lymph nodes. Tumour kinetic parameters (volume transfer constant (Ktrans), extracellular extravascular volume fraction (ve) and plasma volume fraction (vp)) were assessed using the extended Tofts model. Relative changes in studied parameters from baseline to 10 Gy and 40 Gy were calculated. Factors predictive for treatment response were identified by the Firth logistic regression. Spearman's rank correlation coefficient was used to investigate correlations among the parameters.

Results: Responders showed a significant decrease in Ktrans after 10 Gy (median, -50.2%; range, -25.1 to -90.9%; P = 0.047). In addition, decreased ve after 10 Gy (median, -26.9%; range, -76.7 to -126.8%) and increased apparent diffusion coefficient (ADC) after 40 Gy (median, 73.4%; range, 17.8 to -121.6%) were borderline significant (P = 0.066 and P = 0.079, respectively). Positive correlation between Ktrans and ve after 10 Gy (r = 0.823, P<0.05) was noticed.

Conclusion: In HNSCC, early changes in DCE- and DWI-MRI derived parameters appear to predict tumour response before the actual morphologic changes occur.

Disclosure: No significant relationships.

Keywords: Squamous cell head and neck cancer, Concomitant chemoradiotherapy, treatment response, Diffusion-Weighted imaging, Dynamic contrast-enhanced MRI

### SOPS 1.6

**Magnetic resonance texture analysis in the diagnosis of oropharyngeal cancer**

*E. Barabino, S. Caprioli, S. Casella, M. Verda, G. Cittadini; Genoa/Italy*

Short Summary: The aim of our study is to explore the role of Magnetic Resonance Texture Analysis in the Diagnosis of Oropharyngeal Cancer.

Purpose/Objectives: The oropharynx is a challenging district at Computed Tomography (CT) and Magnetic Resonance Imaging (MRI), mostly due to the presence of hyperplastic lymphoid tissue that can disguise malignancies. In some cases CT, MRI, Positron-emission Tomography (PET) and endoscopy, even combined, have disappointing diagnostic performance and could not confirm nor exclude the presence of neoplastic tissue in the oropharynx. Diffusion Weighted Imaging (DWI) in one of the cornerstones in the diagnosis of Head and Neck cancer, but, in the presence of lymphoid tissue of the palatine tonsil and tongue base, restricted diffusion is not a reliable marker. Bhatia et al. demonstrated that Lymphatic Hyperplasia (LH) has even lower Apparent Diffusion Coefficient (ADC) values compared to Squamous Cell Carcinoma (SCC) although a reliable cut-off value was not identified. Texture Analysis is a technique that allows to objectively quantify heterogeneity within a tumor analyzing distribution and spatial interrelationship of pixels. If applied on ADC maps, Texture Analysis could discover new insight about water diffusivity within a tissue. The aim of our study is to investigate if MR Texture Analysis can distinguish a benign condition (LH) from a pathological disease (SCC or Lymphoma) in the oropharynx.

Methods and Materials: We retrospectively evaluated 19 patients that underwent neck MRI at our institution. Nine patients had biopsy-proven T1-T3 Oropharyngeal SCC that affected a tonsil or the base of the tongue. Two patient had Tonsillar Lymphoma (TL). Eight patients with LH that underwent MRI for other indications were enrolled as negative controls. The MR imaging examinations were performed on 1.5T MR scanner (Magnetom Aera, Siemens Healthenieers) with the manufacturer's 8-channel phased-array head and neck coil. A head and neck radiologist, blinded to histological data, manually segmented each structure that showed restricted diffusion in the oropharynx on ADC maps (Figure 1). Ninety-one textural features were extracted using dedicated software (3D Slicer – Radiomics). Statistical Analysis was performed using One way ANOVA or Kruskal-Wallis test to distinguish the three groups. The statistical significance level was set at P = 0.05.

Results: Thirty-six lesions (25 LH, 9 SCC, and 2 TL) were analyzed. Twenty-four textural features were statistically different among the three subgroups (Figure 2).

Conclusion: MR Texture Analysis can accurately differentiate lesions with restricted diffusion in the oropharynx and could be a valuable biomarker in the diagnosis of Oropharyngeal Cancer.

Disclosure: No significant relationships.

Keywords: magnetic resonance imaging, Texture analysis, Apparent diffusion coefficient

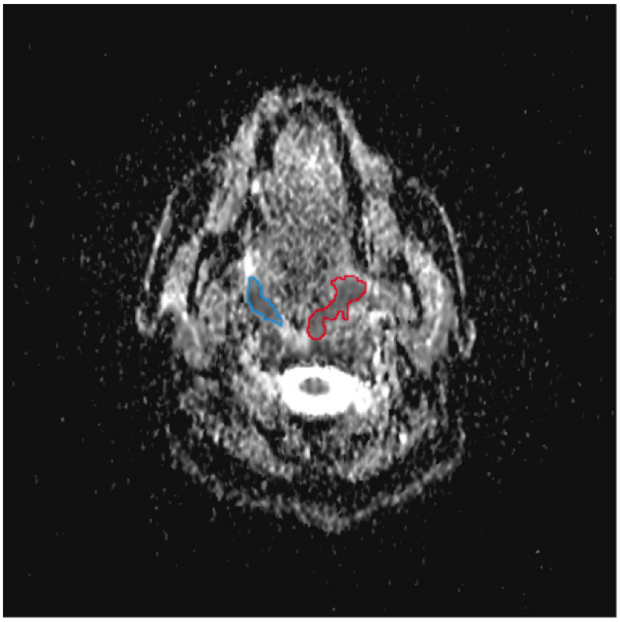

| Features | | Mean | | | P-Value |
|---|---|---|---|---|---|
| Name | Type | LH | SCC | TL | |
| HighGrayLevelEmphasis | gldm | 221,4 | 188,8 | 535,1 | 0,0078 |
| DependenceEntropy | gldm | 6,604 | 6,691 | 7,291 | 0,0172 |
| DepedenceNonUniformity | gldm | 293,9 | 797,3 | 1373 | 0,0098 |
| GrayLevelNonUniformity | gldm | 94,95 | 265,6 | 463,9 | 0,0102 |
| JointAverage | gldm | 12,89 | 12,2 | 21,57 | 0,0179 |
| SumAverage | gldm | 25,77 | 24,41 | 43,13 | 0,0179 |
| Autocorrelation | gldm | 211,8 | 182,3 | 523,9 | 0,008 |
| Idn | gldm | 0,9656 | 0,9686 | 0,9784 | 0,0483 |
| Skewness | first order | 0,289 | 0,6497 | -0,08293 | 0,0296 |
| Energy | first order | 1,02E+09 | 3,85E+09 | 4,98E+09 | 0,005 |
| TotalEnergy | first order | 1,02E+09 | 3,89E+09 | 4,98E+09 | 0,0026 |
| Range | first order | 751,1 | 801,3 | 1133 | 0,047 |
| GrayLevelNonUniformity | glrlm | 60,74 | 158,6 | 260,6 | 0,0055 |
| ShortRunHighGrayLevelEmphasis | glrlm | 197,3 | 165,2 | 428 | 0,0227 |
| RunLengthNonUniformity | glrlm | 663,1 | 1667 | 2926 | 0,0176 |
| RunEntropy | glrlm | 5,407 | 5,526 | 6,065 | 0,0085 |
| HighGrayLevelRunEmphasis | glrlm | 231,4 | 201,9 | 546,8 | 0,0071 |
| GrayLevelVariance | glrlm | 34,49 | 35,57 | 73,36 | 0,0109 |
| SizeZoneNonUniformity | glszm | 113,2 | 198,2 | 358,9 | 0,0111 |
| GrayLeveloneEmphasis | glszm | 18,72 | 37,4 | 47,88 | 0,0333 |
| HighGrayLevelZoneEmphasis | glszm | 271,5 | 266,4 | 591,5 | 0,0095 |
| ZoneEntropy | glszm | 6,457 | 6,759 | 7,605 | 0,0199 |
| Coarseness | ngtdm | 0,03168 | 0,01948 | 0,01177 | 0,0052 |
| Busyness | ngtdm | 0,1189 | 0,1952 | 0,1129 | 0,0424 |

### SOPS 1.7

### Ultrasound guided core biopsies for presurgical staging of oral SCC: Implementing 8th edition AJCC recommendations

*D. Sinha; London/United Kingdom*

Short Summary: The principal change for oral cavity cancers in the 8th edition of the AJCC cancer staging system is the incorporation of the depth invasion (DOI) as an independent criterion for T stage designation.

Purpose/Objectives: The principal change for oral cavity cancers in the 8th edition of the AJCC cancer staging system is the incorporation of the depth of invasion (DOI) as an independent criterion for T stage designation. That the risk of nodal metastasis is directly proportional to DOI of >4mm is well established. This is of importance particularly with regard to the potential need for management of the neck (END v/s SNB). The quality of routine biopsies can make the determination of DOI difficult. In order to facilitate this, we introduce the assessment of the primary oral lesion in conjunction with ultrasound-guided core biopsy as a robust concept for determination of DOI.

Methods and Materials: 20 patients with suspected oral cavity cancer underwent routine ultrasound imaging assessment and the area of interest within the suspected lesion was localized. Transmucosal core biopsy was performed under 4% lidocaine using BioPinceTM full core biopsy instrument. Stroke length was adjusted according to ultrasound findings and tri-axial core biopsies ranging between 9mm-29mm were harvested. No sutures were applied. The core biopsy specimens were histologically evaluated for quality and adequacy of the specimen, presence of disease, DOI, invasive pattern, perineural and lymphovascular invasion.

Results: Male to female ratio was 1:2. Median age 78 years. Distribution based on anatomic area: 70% tongue, 10% gingiva, 10% parotid, 5% tonsil and 5% palate. Up to 59% greater volume of tissue harvest by this method in comparison with traditional core biopsies. All samples obtained were adequate to establish a baseline diagnosis. 50% were positive for SCC. There were no false negative biopsies reported. There were no repeat core biopsies performed. 1 out of the 20 cases had ambiguous histology on core and remained unchanged after final definitive excision. 1 out of 20 cases was downstaged based on core biopsy. No complications post-biopsy were reported. Better allocation of resources as no additional need for formal biopsy in a clinic setting was required.

Conclusion: Ultrasound-guided core biopsies are of good diagnostic quality and can influence the management of the neck and the primary tumour. They are more accurate, cost-effective with minimal complications and false negative rates.

Disclosure: No significant relationships.

Keywords: Depth of invasion, Core biopsy, Ultra sound, Oral Cavity Cancer

### SOPS 1.8

### Evaluation of dynamic contrast-enhanced MRI, diffusion-weighted MRI and 18F-FDG-PET/CT for the detection of unknown primary head and neck squamous cell carcinoma.

*R. Martens; Amsterdam/Netherlands*

Short Summary: Head and neck squamous cell carcinoma (HNSCC) could present with cervical lymph node metastasis of unknown primary tumor (UPT). Improving occult primary tumor detection may aid in providing more targeted treatment for patients.

Purpose/Objectives: This study assessed the accuracy of qualitative DCE, DWI and $^{18}$F-FDG-PET/CT imaging for unknown primary tumor detection.

Methods and Materials: Between July 2014 and February 2019, patients were prospectively included at our tertiary referral center for head and neck cancer, with histologically proven cervical squamous cell carcinoma (SCC) lymph node metastasis of UPT and underwent $^{18}$F-FDG-PET/CT, DWI- and DCE-MRI. For comparison also patients with overt primary tumors were included. Qualitative assessment was performed by two observers per modality. Sensitivity and specificity were calculated for each 5-, 3- and 2-pointscale score, of which conservative and sensitive scoring strategies were distracted. The interobserver agreement was calculated using a linear weighted Cohen's Kappa and specific agreement.

Results: In total 64 patients were included (46 male (71.9%); median age 62 years, range 45-81 years), including 35 overt primary tumors and 29 UPT's. The highest interobserver and agreement in positive-scored lesions was for the 5-point-scale in per patient and per lesion analysis. The sensitive scoring in a per-patient analysis resulted in a 95.0% (95%CI=75.1-99.9%) sensitivity for DWI, 95.0% (95%CI=75.1-99.9%) for DCE and 100% (95%CI=83.2-100%) for $^{18}$F-FDG-PET/CT. The per-patient sensitivity of DWI-/DCE-MRI-combination was similar to 18F-FDG-PET/CT (100%, 95%CI=83.2-100%), whereas specificity was non-significantly lower 11.1% (95%CI=32.9-82.5%) versus (22.2% (2.8-60%). The DWI/DCE-MRI-combination, per-lesion, using a 5-point-scale resulted in a non-significant higher sensitivity for DWI/DCE-MRI (95.0%, 95%CI=75.1-99.9%) than $^{18}$F-FDG-PET/CT (90.0%, 95%CI=68.3-98.8%).

Conclusion: The accuracy and agreement of DCE, DWI and $^{18}$F-FDG-PET/CT to detect occult primary tumors in patients with cervical squamous cell carcinoma metastases of unknown primary tumor was high. Combined use of DCE- and DWI-MRI yield similar or higher accuracy than $^{18}$F-FDG-PET/CT.

Disclosure: No significant relationships.

Keywords: Unknown Primary, squamous cell carcinoma, head and neck, Diffusion-weighted MRI, $^{18}$F-FDG-PET/CT, Dynamic contrast-enhanced MRI

### SOPS 1.9

### Prediction of tumor grade and nodal status in oropharyngeal and oral cavity squamous cell carcinoma: a quantitative radiomics study applied to primary tumor lesions

*A. Stanzione, V. Romeo, C. Ricciardi, R. Cuocolo, L. Ugga, F. Verde, S. Cocozza, A. Elefante, S. Staibano, A. Brunetti; Naples/Italy*

Short Summary: Contrast-enhanced CT images of 40 patients with histologically proven oropharynx (OP) and oral-cavity (OC) squamous-cell-carcinoma (SCC) were post-processed to extract texture-analysis (TA) quantitative features from primary tumor lesions (PTLs) for the prediction of tumor grade (TG) and nodal status (NS). The Hold-out, wrapper method and a 10-fold cross-validation were then applied. For the prediction of TG, the best accuracy (92.9%) was achieved by Naïve Bayes (NB), bagging of NB and K Nearest Neighbor (KNN). For the prediction of NS, J48, NB, bagging of NB and boosting of J48 overcame the accuracy of 90.0%. A radiomic approach employing TA quantitative features applied to PTLs may be reliable in predicting TG and NS in OP and OC SCC patients.

Purpose/Objectives: We aimed to investigate whether a radiomic approach employing texture analysis (TA) quantitative features applied to primary tumor lesions (PTLs) could be effective in the prediction of tumor grade (TG) and nodal status (NS) in oropharyngeal (OP) and oral cavity (OC) squamous cell carcinoma (SCC) patients.

Methods and Materials: Contrast-enhanced CT images of 40 patients with OP and OC SCC, histologically classified as G2 (n=13) and G3 (n=27) as well as N0 (n=19) and N+ (n=21), were post-processed to extract TA quantitative features through manual segmentation of PTLs. A synthetic minority oversample technique was applied to balance the dataset, increasing the number of records till 50 in TG and 42 in NS classification. A hold-out was then performed to split the population in a training (75%) and a test set (25%). Subsequently, the wrapper method and a 10-fold cross-validation were applied in order to find the best subset of features to achieve the highest accuracy in the prediction of TG and NS.

Results: Concerning the prediction of TG, the best accuracy (92.9%) was achieved by Naïve Bayes (NB), bagging of NB and K Nearest Neighbor (KNN), while the highest AUCROC was obtained by NB, bagging of J48, KNN and boosting of J48 that were all > 90%. For the prediction of NS, J48, NB, bagging of NB and boosting of J48 overcame the accuracy of 90.0%, while the highest AUCROC was achieved by J48, KNN, boosting of J48 and NB (0.900) as well as bagging of J48 (0.917).

Conclusion: A radiomic approach employing TA quantitative features applied to PTLs may represent a reliable tool in predicting TG and NS in patients with OC and OP, potentially providing valuable information to establish the most appropriate treatment and identify high-risk patients.

Disclosure: No significant relationships.

Keywords: Radiomics, Texture analysis, Machine learning, Squamous-cell carcinoma, Oropharynx, oral cavity

### SOPS 1.10

### Diagnostic accuracy of magnetic resonance imaging in the detection of bone invasion from oral cavity cancer

*R. Taravella, A. Cordova, M. Attanasio, V.G. Genova, G. Campisi, G. Rinaldi, V. Rodolico, D. Matta, N. Feo, A. Lo Casto; Palermo/Italy*

Short Summary: Suspicion of bone infiltration from oral cavity cancer (OCC) influences perioperative strategy, requiring bone resection and recostruction in some cases, with a considerable impact on outcome and quality of life.

Purpose/Objectives: To retrospectively evaluate diagnostic accuracy of preoperative MRI in the assessment of bone invasion by OCC, having histopathology as standard of reference.

Methods and Materials: A population of patients studied by MRI between March 2013 and April 2019, with histopathologically confirmed diagnosis of OCC was retrospectively analyzed.All patients were studied on preoperative MRI. Inclusion criteria were: surgical procedure, preoperative MRI with an imaging-to-surgery time interval of 0-4 weeks and histophatological examination performed in our Istitution. Patients who didn't performed MRI or histopathological confirmation,who underwent preoperative treatments with radio-chemo-therapy or partial resections were excluded. Surgical and pathological records were matched with MRI reports, retrieved by Radiology Department RIS-PACS. A population of 99 patients satisfying inclusion criteria was identified. All MR exams were performed with the same 1.5T superconductive system. The study consisted in spin-echo (TSE) T1 sequences with and without fat-saturation and intravenous administration of gadopentate dimeglumine (Gd-DPTA) at 0.1ml/kg and T2, STIR, DWI, FFE 3D T1 fat saturated sequences with a 1-to-4 millimeter thick-slice on axial, coronal, sagittal plane. MRI diagnostic criteria used for evaluation of bone invasion were:lack of typical hypointense signal of cortical bone on T1 and T2, replaced by adjacent tumor signal intensity (to assess cortical invasion);evidence of hyperintense signal of bone marrow adjacent to tumor on STIR and T1 enhanced sequences (to demonstrate marrow involvement). All patients underwent surgical resection with marginal, segmental mandibulectomy, infrastructure maxillectomy or excisional biopsy for localized lesions. ROC curves were used for statistical analysis.

Results: Histological tumor types were: 96% squamous cell carcinoma (16% low-to-high dysplasia and in situ carcinoma, 80% carcinoma); 1%oral salivary duct in situ carcinoma; 1% mucoepidermoid oral salivary gland carcinoma; 1% spindle cell carcinoma; 1% polymorphous low-grade adenocarcinoma. OCC anatomical sites were: 41.4% oral mucosa, 2.1% soft palate, 2% hard palate, 37.4% tongue (anterior two-thirds), 2% floor of mouth, 1% retromolare trigone, 2% lips, 12.1% overlapping sites of several anatomic locations. Patients were classified into a 5 groups-severity scale in ROC curves (Figure 1) with 4 cut-offs: low-to-high localized dysplasia and precancerous lesions; OCC without bone invasion; OCC with superficial focal erosions of mandibular bone without deep invasion; OCC with deep invasion of mandibular bone; OCC with deep invasion of both mandibular and jaw bone. MRI accuracy (Table a) and Kendall's τ concordance index (0.77) for ordinal data showed a good degree of agreement between MRI and histopathology.

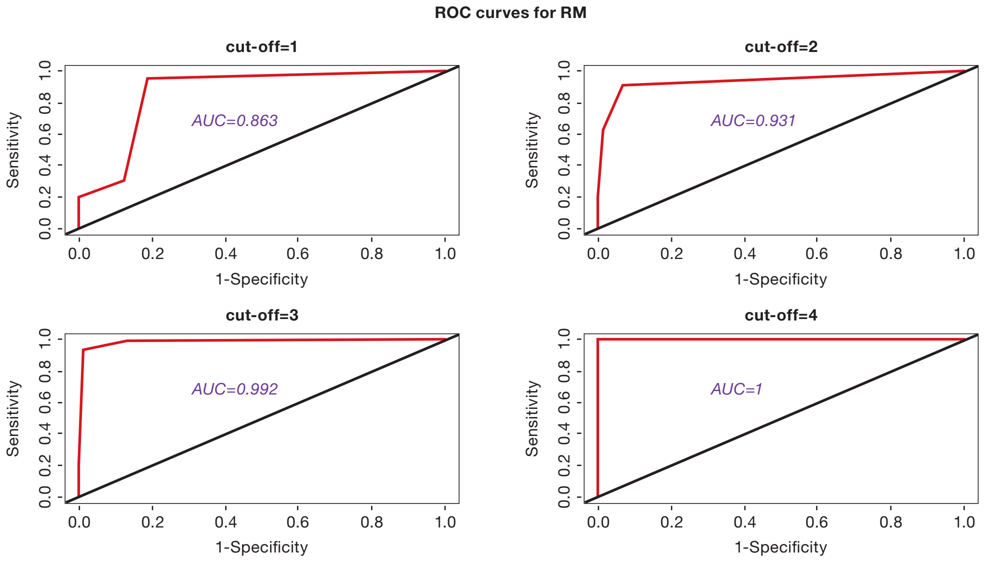

*Table a*

| | cut-off=1 | cut-off=2 | cut-off=3 | cut-off=4 |
|---|---|---|---|---|
| **Specificity (SP)** | 0.76 | 0.97 | 0.98 | 0.97 |
| **Sensitivity (SE)** | 0.96 | 0.81 | 0.94 | 1 |
| **Positive Predictive Value (PPV)** | 0.95 | 0.91 | 0.94 | 1 |
| **Negative Predictive Value (NPV)** | 0.81 | 0.93 | 0.98 | 1 |

Conclusion: Preoperative MRI is accurate in predicting bone involvement by OCC.

Disclosure: No significant relationships.

Keywords: ROC curves, Oral Cavity Cancer, magnetic resonance imaging, bone invasion

### SOPS 1.11

### Diagnostic accuracy and prognostic value of tumor depth of invasion detected with magnetic resonance imaging in oral cavity cancer

*R. Taravella, A. Cordova, V. Restivo, C. Costantino, G. Campisi, G. Rinaldi, V. Rodolico, D. Matta, N. Feo, A. Lo Casto; Palermo/Italy*

Short Summary: According to 8th AJCC TNM Staging,patients with oral cavity cancer (OCC) should be classified into 4 T-staging groups, using tumor depth of invasion (DOI) parameter.

Purpose/Objectives: To retrospectively evaluate diagnostic accuracy of preoperative MRI in the assessment of radiologic DOI (rDOI) by OCC, having histopathology (with pathologic DOI, pDOI) as standard of reference. To determine prognostic value of rDOI-pDOI correlation.

Methods and Materials: A population of patients studied by MRI between March 2013 and April 2019, with histopathological diagnosis of OCC was retrospectively analyzed. All patients were studied on MRI. Inclusion criteria were: surgical procedure, preoperative MRI with an imaging-to-surgery time interval of 0-4 weeks and histophatological examination performed in our Istitution. Patients who didn't performed MRI or histopathological confirmation, who underwent preoperative treatments with radio-chemo-therapy or partial resections were excluded. Surgical and pathological records were matched with MRI reports,retrieved by Radiology Department RIS-PACS. A population of 99 patients satisfying inclusion criteria was identified. Pathologists measured pDOI on specimens from the basement membrane of adjacent normal epithelium to the deepest extent of tumor. All MR exams were performed with the same 1.5T superconductive system. MRI consisted in spin-echo (TSE) T1 sequences with and without fat-saturation and intravenous administration of gadopentate dimeglumine (Gd-DPTA) at 0.1ml/kg and T2, STIR, DWI, FFE 3D T1 fat saturated sequences with a 1-to-4 millimeter thick-slice on axial, coronal, sagittal plane. rDOI was measured in nonenhanced T1 and T2 sequences(best delineating tumor margins), in a plane perpendicular to the mucosal surface. All patients underwent surgical resection with marginal, segmental mandibulectomy, infrastructure maxillectomy or excisional biopsy for localized lesions. Hazard Ratio(HR) was calculated with Cox regression-Breslow method for ties.

Results: Histological tumor types were: 96% squamous cell carcinoma (16% low-to-high dysplasia and in situ carcinoma, 80% carcinoma); 1% oral salivary duct in situ carcinoma; 1% mucoepidermoid oral salivary gland carcinoma; 1% spindle cell carcinoma; 1% polymorphous low-grade adenocarcinoma. OCC anatomical sites were: 41.4% oral mucosa, 2.1% soft palate, 2% hard palate, 37.4% tongue (anterior two-thirds), 2% floor of mouth, 1% retromolare trigone, 2% lips, 12.1% overlapping sites of several anatomic locations. MRI accuracy in the detection of rDOI for the 4 TNM T-staging groups(Table a) and rDOI-pDOI correlation (Figure 1) are high. Table b shows HR of rDOI. Further analysis are in progress to completely clarify prognostic value of rDOI.

*Table a*

| | T1 | T2 | T3 | T4 |
|---|---|---|---|---|
| **Sensitivity (SE)** | 89% | 88% | 96% | 94% |
| **Specificity (SP)** | 97% | 100% | 99% | 99% |
| **Positive Predictive Value (PPV)** | 92% | 100% | 96% | 94% |
| **Negative Predictive Value (NPV)** | 96% | 98% | 99% | 99% |
| **Accuracy** | 95% | 98% | 98% | 98% |

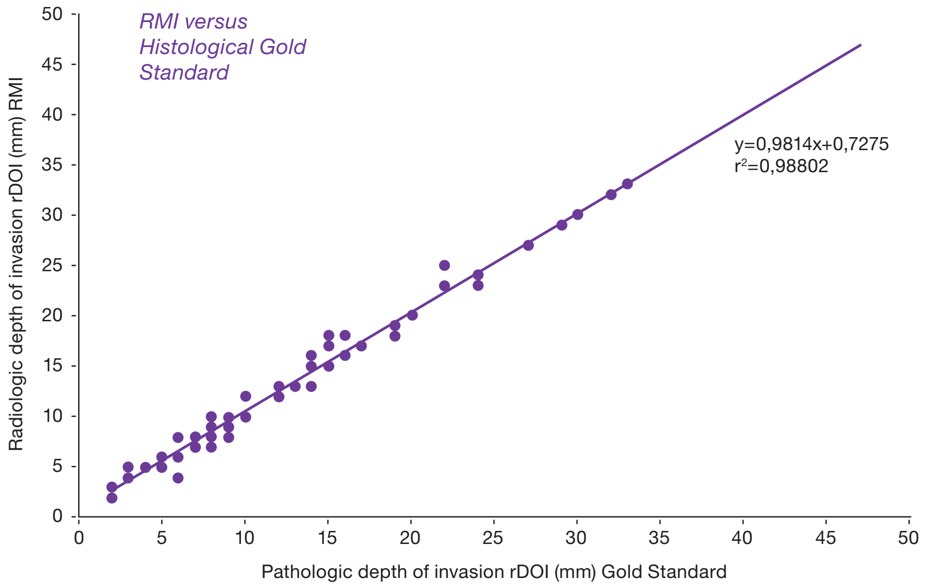

*Table b*

| | HR | [95% Confidence Interval] |
|---|---|---|
| **Death** | 3.28 | [1.54,6.99] |
| **Gender** | 0.95 | [0.59,1.52] |
| **Age** | 1 | [0.98,1.02] |
| **rDOI:** | | |
| **T1** | 0.53 | [0.28,1.03] |
| **T2** | 0.55 | [0.27,1.14] |
| **T3** | 0.46 | [0.23,0.95] |
| **T4** | 0.40 | [0.19,0.83] |

Conclusion: rDOI is accurate in predicting tumor DOI with good rDOI-pDOI correlation and HR.
Disclosure: No significant relationships.
Keywords: Oral Cavity Cancer, magnetic resonance imaging, tumor depth of invasion, Cox regression-Breslow method for ties

### SOPS 1.12

### Lymphoma of the head and the neck: our systematic review

*A. Modoni, M. Magli, F. Franco, T. Magli; Martina Franca/Italy*

Short Summary: The Lymphomas of the head and the neck are a heterogeneous group of malignant tumours of the haematopoietic system and are characterized by the aberrant proliferation of mature lymphoid cells or their precursors Lymphomas can be divided into two major entities: Hodgkin's lymphoma (HL) and non-Hodgkin's lymphoma (NHL). Over 20 different subtypes of NHL have been classified according to the specific subtype of lymphoid cells involved. Non-Hodgkin's lymphoma is diagnosed in extranodal sites in 40% of cases, and the head and neck region is the second most affected, with an incidence of 11–33%, while HL has a very low incidence in extranodal sites (2–3%). Method: From 2010 to 2018, all Patients (vn= 120) with histologically defined H&N lymphoma from our clinic were evaluated. Results: This study identified 120 patients with H&N lymphoma comprising 93 non-Hodgkin lymphomas (NHL) and 27 Hodgkin lymphomas (HL). Among NHL there were 40 indolent (iNHL), 47 aggressive (aNHL), 6 highly aggressive NHL and further 27 HL. Patients with highly aggressive NHL and HL were significantly younger (p < 0.0001). In oure experience non Hodgkin lynphoma in extra nodal sites
Purpose/Objectives: The objective of the study is to evaluate all sites of head and neck lymphomas combining clinical signs and imaging to differentiate Hodgkin and non-Hodgkin lymphomas from the remaining neoplastic lesions of these sites.
Methods and Materials: From 2010 to 2018, all Patients (vn= 120) with histologically defined H&N lymphoma from our clinic were evaluated.
Results: This study identified 120 patients with H&N lymphoma comprising 93 non-Hodgkin lymphomas (NHL) and 27 Hodgkin lymphomas (HL). Among NHL there were 40 indolent (iNHL), 47 aggressive (aNHL), 6 highly aggressive NHL and further 27 HL. Patients with highly aggressive NHL and HL were significantly younger (p < 0.0001). In oure experience non Hodgkin lynphoma in extra nodal sites
Conclusion: early diagnosis and subsequent immediate treatment, especially in aggressive ones, is important for proper therapy. the biopsia in these locations
Disclosure: No significant relationships.
Keywords: head and neck, IMAGING LYMPHOMAS, BIOPSY, LYMPHOMAS

### SOPS 1.13

### Dual source dual energy computer tomography in submandibular sialolithiasis; reliability and radiation burden

*G.G. Pulickal, D. Singh; Singapore/Singapore*

Short Summary: Sialolithiasis is an often encountered problem in Head & Neck imaging. Non contrast CT (NCCT) is usually used for detection and pre-operative assessment. NCCT has its limitations as does Contrast enhanced CT (CECT) by itself. A combined study of NCCT & CECT provides a complete assessment but at a high radiation. Single phase Contrast enhanced Dual energy CT with reconstructed virtual non contrast (VNC) images could provide an elegant and simple solution.
Purpose/Objectives: This study aims to compare the diagnostic accuracy of virtual non contrast images derived from dual source dual energy contrast enhanced computer tomography (CT) to standard non contrast CT images, for evaluation of sialolithiasis.
Methods and Materials: Retrospective analysis of all dual energy CT studies of the neck performed over the preceding five years were reviewed for submandibular gland calculi. Only studies that had undergone both non contrast CT and contrast enhanced CT as part of the same evaluation were included in this study. This yielded a total of 30 cases. Virtual non contrast images were derived from the dual energy data set and compared to the true non contrast images by two separate radiologist who assessed the total number of calculi encountered, their location, largest dimension and the density of the calculi. The radiation burden incurred for true non contrast, virtual non contrast and total was calculated as well.

Results: Our analysis revealed that measuring of stone size showed good inter-observer agreement. The average stone size on VNC was 7.9 +/- 5.8 mm and 8.4 +/- 5.7 mm on TNC (ranging from 2.0 mm to 31.8 mm), the difference being statistically insignificant. The average stone attenuation varied considerably (p <0.01) being 494.8 +/- 187.5 for VNC and 924.4 +/- 374.9 for TNC. The average radiation dose for a dual phase intravenous contrast enhanced CT study was 23.13 mGY ($CTDI_{vol}$). The mean dose was 10.93 mGY for the true non contrast phase, thereby, suggesting a 47.25 % reduction in administered radiation dose when a single phase contrast enhanced study with virtual non contrast reconstructions is performed.
Conclusion: Virtual non contrast images derived from dual source dual energy CT scans of the neck provides accurate assessment of sialolithiasis akin to conventional CT imaging protocols at only a fraction of the radiation dosage.
Disclosure: No significant relationships.
Keywords: Sialolithiasis, Virtual non contrast, Radiation Burden

### SOPS 1.14

### The role of the texture analysis in the characterization of parotid salivary gland lesions: a study on MR diffusion weighted imaging

*C. Nardi, M. Tomei, M. Pietragalla, F. Mungai, L. Calistri, S. Colagrande; Florence/Italy*

Short Summary: Texture Analysis is a method applied to digital images to extract “radiomic features” taking into account a spatial neighborhood of pixels in a region of interest. Recently, some articles assessed clinical applications of the texture analysis in head and neck cancers, but its role in the characterization of parotid gland lesions is still missing.
Purpose/Objectives: To evaluate the usefulness of the MR Diffusion weighted Imaging (DwI) texture analysis both in characterizing of pleomorphic adenoma, Warthin tumor, epithelial malignancy and lymphoma and in distinguishing between benign and malignant parotid lesions.
Methods and Materials: 105 patients with lesions of parotid glands underwent head and neck MRI (1.5 T Magnetom Aera, Siemens). The patients/lesions matched our inclusion criteria (Table 1) were 42. DwI with two b values (50 and 800 s/mm$^2$) was acquired and apparent diffusion coefficient (ADC) map images were obteined to calculate the volume of each lesion. Fourty-one texture features were calculated (Table 2). ANOVA test was used to estimate both the distinction between benign and malignant lesions and the texture feature differences among pleomorphic adenoma, Warthin tumor, lymphoma, and epithelial malignancy. P-value≤0.01 was considered to be statistically significant. A cut-off value defined by the ROC curve analysis was found for each statistically significant texture parameter. Sensitivity, specificity, positive predictive value (PPV), negative predictive value (NPV), and diagnostic accuracy were obtained for each texture parameter with area under the curve (AUC) >0.5. The agreement between each texture parameter and the reference standard represented by histology was calculated using the Cohen’s kappa coefficient.
Results: The mean kappa value of the statistically significant texture parameters was 0.61, 0.34, 0.26, 0.17, and 0.48 for lymphoma, epithelial malignancy, Warthin tumor, pleomorphic adenoma, and benign lesions vs. malignant lesions, respectively.
Energy, homogeneity, long run emphasis, long-run low gray-level emphasis, and long zone emphasis (LZE) were significant parameters in the characterization of lymphoma. All of them showed moderate or substantial agreement with histology (K=0.50-0.73).
In the characterization of epithelial malignancies and in distinguishing between benign and malignant lesions, LZE showed almost substantial (K=0.57) and substantial agreement (K=0.61) respectively.
K values between 0.07 and 0.40 were found in all parameters analyzed for Warthin tumor and pleomorphic adenoma.
Conclusion: Texture Analysis showed a moderate or substantial agreement with histology in all the parameters examined for lymphoma, whereas LZE was the reference parameter in characterizing of epithelial malignancies and in distinguishing between benign and malignant lesions. Warthin tumour and pleomorphic adenoma were not accurately recognized.
Disclosure: No significant relationships.
Keywords: Salivary glands, Parotid cancer, Texture analysis, magnetic resonance imaging, Diffusion Weighted Imaging

### SOPS 1.15

### IVIM and DCE-MRI features to differentiate benign and malignant parotid tumors

*F. Patella[1], M. Sansone2, G. Franceschelli[1], R. Fusco[2], M. Fusco[1], L. Tofanelli[1], G. Nicolino[1], G. Buccimazza[1], M. Petrillo[1], G. Carrafiello[1]; [1]Milan/Italy, [2]Naples/Italy*

Short Summary: Parotid tumors still represent a diagnostic challenge for both cytology and radiology, because of the huge variety of hystotypes and frequent overlapping of both cytological and imaging features between them. In this pilot study, we propose the use of IVIM as well as DCE-MRI parameters, including both quantitative and semiquantitative features, to differentiate the most frequent benign parotid tumors (Warthin tumors and pleomorphic adenomas) from malignant tumors. Our hypothesis is that the additional employ of a wider group of MRI features, could integrate the information provided by ADC and time-intensity curves, which are commonly used in everyday practice, increasing MR accuracy in differential diagnosis of salivary gland neoplasias.
Purpose/Objectives: The purpose of this pilot study was to characterize parotid tumors and to distinguish Warthin tumors (WT) and Pleomorphic Adenomas (PA) from malignant tumors (MT), by correlating functional MRI parameters with pathology.

Methods and Materials: A total of 42 histopathologically proven parotid tumors (17 WT, 17 PA and 8 MT) in 35 consecutive enrolled patients (20 women and 15 men) were included in this study. For both DCE-MRI and DW-MRI model-free and model-based parameters were computed voxel-by-voxel on manually segmented volumes of interest (VOIs) around the lesions. The average values of the following features were extracted: Ktrans, kep, vp, MRE, TTP, WIS, WOS, f, D, D*. The Nemenyi test was used to compare the parameters in WT, PA and MT.
Results: As regards PA and WT, the Nemenyi test revealed that significant difference (p<0,05) could be found for TTP.av, Ktrans.av, Dstar.av and D.av. With respect to PA and MT, significant difference at the Nemenyi Test was identified for Ktrans.av, Dstar.av. Importantly, WT and MT presented significant difference only for just one parameter: WIS.av. Figure 1 details these results. Notably, MT showed the highest values of WIS.av, while PA presented the highest values of D.av and TTP.av.

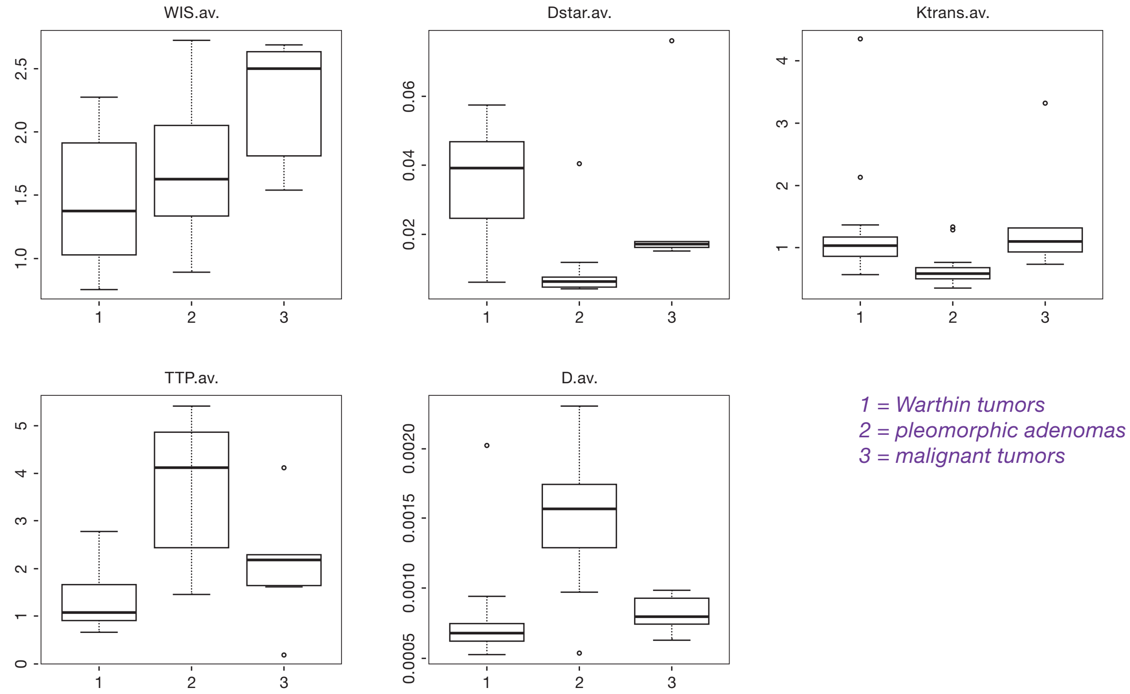

Conclusion: DWI and DCE-MRI features may help WT, PA and MT differential diagnosis.
Disclosure: No significant relationships.
Keywords: MRI, DCE-MRI, IVIM, parotid tumors

### SOPS 1.16

### The lymphoid organs condition in children with juvenile parotitis

*A. Nadtochiy, O. Vozgoment, E. Kostenko; Moscow/Russian Federation*

Short Summary: Juvenile parotitis (JP) is the most common inflammatory disease of parotid glands in children with persistent course with exacerbations and remissions and great difficulties in treatment.
Purpose/Objectives: Determination of lymphoid organs condition in children with JP based on complex ultrasound examination (US).
Methods and Materials: 50 patients 3.5-10 years (29 boys, 21 girls) with different stages of JP were undergone US of parotid glands (PG), neck and mesentery lymph nodes (LN), spleen. Evaluated LN parameters: linear size and L/S ratio, cortical layer thickness, gate LN artery peak velocity (Vmax). Evaluated spleen parameters: lymphoid follicles number, size and distribution; spleen mass coefficient (SMC) – ratio of the spleen mass and the body weight. The relationships of LN and SMC parameters were analyzed mathematically.
Results: Bilateral JP was detected in 39 (78%) patients: the activity of the inflammatory process was the same in both PG in 12 (30.8%) patients. LN analysis: - jugulodigastral LN: linear size 24.7mm to 31.5mm (27.4 + 1.2mm); cortical layer thickness: 1.1 - 1.5 mm (1.3 + 0.14 mm); Vmax: 11.7-18.5 (14.5 + 2.1 cm/s). - mesenteric LN: linear size: 11.7mm to 15.6mm (14.0 + 0.9mm); cortical layer thickness: 0,7 - 1,0mm (0,85 + 0,1mm); Vmax: 4.1-8.5 (6.4 + 1.7 cm/s). - L/S ratio of cervical and mesenteric LN: 2.1: 1 - 3.2: 1 (2.74 + 0.31); Spleen analysis: - SMC ranged from 3.9 to 5.7 (mean group = 4.4 ± 0.4) (norm = 2-4). - lymphoid follicles: number and size increased together with SMC. Mathematical analysis confirmed the direct relationship of LN and SMC parameters.
Conclusion: The systemic lymphoid organs (cervical and mesenteric LN, spleen) hyperplasia in JP patients was revealed. This indicates the immunocompromise of these patients. The obtained data show the important role of the immune system in JP pathogenesis.
Disclosure: No significant relationships.
Keywords: Spleen mass coefficient, Ultrasound of Lymphoid organs, Juvenile parotitis, Systemic Lymphoid organs hyperplasia, Spleen hyperplasia

### SCIENTIFIC ORAL PRESENTATION SESSION: EAR/ORBIT, SKULL BASE, MISCELLANEOUS

### SOPS 2.1

### Noise reduction and image quality in ultra-high-resolution computed tomography of the temporal bone using advanced modeled iterative reconstruction

*J.-M. Hempel, M. Bongers, K. Braun, U. Ernemann, G. Bier; Tübingen/Germany*

Short Summary: The advanced modeled iterative reconstruction (ADMIRE) algorithm significantly improves image quality and reduces noise on temporal bone ultra-high resolution computed tomography cans. Thus, it allows for substantial dose reduction. For effective noise reduction an ADMIRE strength level of at least A2 is recommended. In the subjective analysis, ADMIRE strength level of A3 performed best in delineating temporal bone substructures.

Purpose/Objectives: To assess the potential of advanced modeled iterative reconstruction algorithm (ADMIRE) in ultra-high resolution (UHR) computed tomography (CT) of the temporal bone.

Methods and Materials: Forty-four patients who underwent UHR CT of the temporal bone using z-axis ultra-high-resolution protocol were retrospectively selected for analysis. Images were reconstructed using filtered back projection (FBP) and ADMIRE with multiple strength levels. Regions of interest were drawn in the posterior fossa and petrous bone. The average density (in Hounsfield units, HU) and the image noise (standard deviation of density values) were extracted. The signal to noise and contrast to noise ratios (SNR and CNR) were then calculated. Additionally, a subjective qualitative analysis was performed using a five-point Likert scale. The potential dose reduction was extrapolated from objective analysis and confirmed in an additional phantom study.

Results: The image noise was significantly lower, and the SNR and CNR were significantly higher in ADMIRE reconstructions levels A2–A5 than in FBP ($p < 0.001$, respectively).

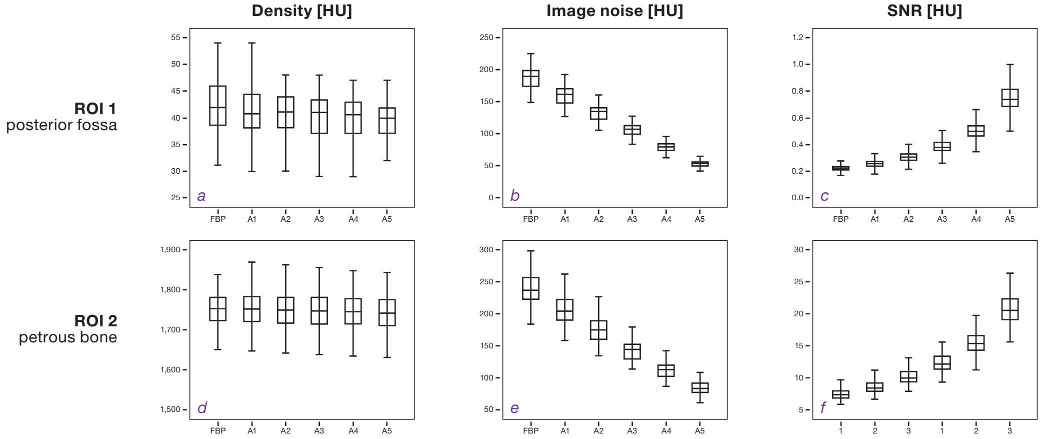

Subjective image quality was significantly higher in ADMIRE levels A2-A5 than in FBP ($p < 0.001$), and it was rated best in ADMIRE level A3. A dose reduction of 40% was feasible while maintaining image quality.

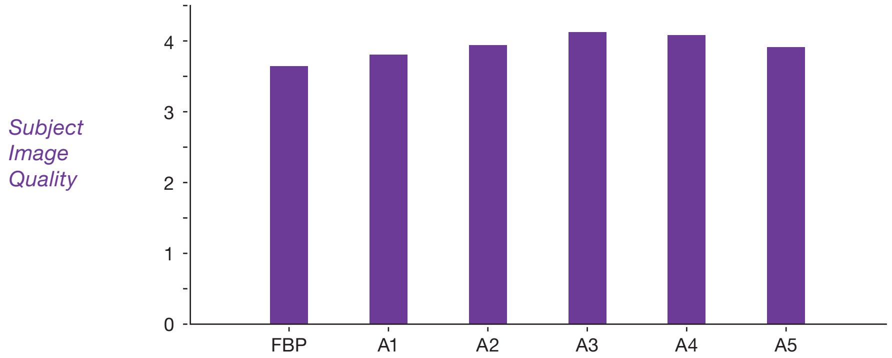

Conclusion: The ADMIRE reconstruction algorithm significantly improves image quality and reduces noise on temporal bone UHR CT scans. Thus, it allows for substantial dose reduction. For effective noise reduction an ADMIRE strength level of at least A2 is recommended. In the subjective analysis, ADMIRE strength level of A3 performed best in delineating temporal bone substructures.

Disclosure: No significant relationships.

Keywords: temporal bone, iterative reconstruction, advanced modeled iterative reconstruction, ADMIRE, computed tomography

### SOPS 2.2

### Black bone MRI as a novel technique to aid the pre-operative planning of cochlear implantation

*S. Connor, I. Pai, H. Barnsley; London/United Kingdom*

Short Summary: We evaluated the ability of black bone MRI to delineate the mastoid facial nerve canal and the corda tympani, which are the key anatomical landmarks for a posterior tympanotomy in 25 patients. The black bone MRI sequence clearly demonstrated the facial nerve in all cases and the chorda tympani in 72% of cases, with the course corresponding to that demonstrated on CT in the majority of cases.

Purpose/Objectives: We evaluated the ability of black bone MRI to delineate the mastoid facial nerve canal and the chorda tympani, which are the key anatomical landmarks for a posterior tympanotomy.

Methods and Materials: Two observers reviewed the black bone MRI studies followed by the CT studies in this prospective study, with respect to the visibility of the mastoid facial nerve canal and the chorda tympani (n=50). In each case, black bone MRI and and equivalent CT "visibility scores" for the mastoid facial nerve and the chorda tympani were recorded. A "corresponding score" (0-3) was then recorded according to whether the nerves demonstrated on MRI corresponded to the paths of the nerves on CT.

Results: When evaluating the black bone MRI, the nerves were felt to be demonstrated on all or the majority of sections and were clearly demonstrated relative to background (scores 2/3) in 50/50 (facial nerve) and 36/50 (chorda tympani) cases, with mean "visibility scores" of 2.82 (facial nerve) and 1.9 (chorda tympani). The equivalent scores for CT were 50/50 (facial nerve) and 40/50 (chorda tympani) with mean "visibility scores" of 2.88 (facial nerve) and 2.26 (chorda tympani). Following CT review, all the nerves identified on MRI corresponded to the path of the nerves on CT (scores 2/3) in 50/50 (facial nerve) and 39/40 (chorda tympani) of cases, with mean "corresponding scores" of 2.88 (facial nerve) and 2.28 (chorda tympani). MIP reconstructions provide a useful pictorial representation of the posterior tympanotomy surgical approach and visibility of the round window.

Conclusion: Using a dedicated "black bone" sequence it is possible to identify the mastoid facial nerve canal on the majority of sections in all patients, and the chorda tympani on the majority of sections in 72% of adult patients. The chorda tympani identified almost always corresponded to that identified with CT. Black bone MRI may be a useful adjunct to pre-cochlear implant MRI protocols in those centres where CT is not routinely performed, in order to provide further information on the trajectory of the proposed posterior tympanotomy.

Disclosure: No significant relationships.

Keywords: cochlear implant, temporal bone, magnetic resonance imaging

### SOPS 2.3

### In vivo detection of cochlear implant-induced new bone formation by ultra high-resolution CT and its clinical relevance

*F. Lucev[1], F. Heutink[2], W. Huinck[2], P. Vart[2], W.J. Woude, Van Der[2], T. Meulman3, L. Mens[2], E. Mylanus[2], B. Verbist[2,3] [1]Pavia/Italy, [2]Nijmegen/Netherlands, [3]Leiden/Netherlands*

Short Summary: In this study we propose a radiologic non invasive method to assess New Bone Formation in the cochlea after cochlear implantation (CI), which is associated with poorer hearing outcome.

Purpose/Objectives: The indications for cochlear implantation have become widespread over years with an increasing number of recipients with residual hearing. Surgical insertion of cochlear implant electrodes may induce delayed changes within the cochlea due to insertion trauma such as inflammation, fibrosis and new bone formation (NBF), with possible impact on loss of residual hearing and hearing outcome. Previous histopathologic temporal bone studies reported contrasting effects of NBF on audiometric outcomes. To our knowledge, this is the first radiologic study using an ULTRA high-resolution CT-scan to visually assess NBF, relate it with electrode scalar position and trajectory and, secondly, to investigate the influence on residual hearing.

Methods and Materials: Participants were 125 postlingually deafened adult users of the Cochlear device with a Contour Advanced or a Slim Straight electrode. CT-scans were conducted on an ULTRA high-resolution CT (Precision Aquilion, Canon Medical Systems, Otawara, Japan) at least one year post-implantation. The electrode position variables were analysed on cochlear multiplanar reconstructions. The assessed variables were 1) electrode scalar trajectory including scalar translocation, 2) electrode insertion angle and 3) wrapping factor (electrode proximity to the modiolus). NBF around the electrode was determined and visually scored per electrode contact on midmodiolar reconstructions by two radiologists independently. Preoperative and postoperative residual hearing was measured using pure-tone audiometry.

Results: The results showed NBF on one or more contacts in 60,3% of the patients. Of all evaluated contacts (n=2750), 13.1% were found to be impacted by NBF. In 91,4% of all affected electrodes, NBF was located around the nine most basal contacts. Inter-observer reliability of the NBF scores of the two radiologists was 85%. Analysis showed that surgical factors – e.g. electrode type and surgical technique, were correlated with the risk for NBF. Participants with scalar tympani position and postoperative residual hearing below 100dB were grouped into two groups based on the presence of NBF. The group with one or more contacts with NBF had significant higher loss of long-term residual hearing compared to the group without formation of NBF (p=0.02), respectively, 22.2 dB and 8.8 dB.

Conclusion: The present study showed that ULTRA high-resolution CT-scan provides a reliable image to judge the occurrence of New Bone Formation. NBF is clinically relevant as it showed to negatively affect the preservation of long-term residual hearing.

Disclosure: No significant relationships.

Keywords: cochlear implant, electrode insertion trauma, sensorineural hearing loss, hearing preservation, new bone formation

### SOPS 2.4

### Comparing cohlear nerve dimensions between the patients with postlingual profound hearing loss and control group with magnetic resonance imaging

*M.B. Eser, B. Atalay, M.T. Kalcioğlu, U.P. Orhan Söylemez; Istanbul/Turkey*

Short Summary: This study is about cochlear nerve dimensions in MRI for evaluating the differences between patients with profound hearing loss and normal people.

Purpose/Objectives: To investigate and compare the dimensions of the cochlear nerve (CN) among individuals profound sensorineural profound hearing loss prior to Cochlear implantation (CI) and normal individuals. And also to investigate the correlation between the duration of the hearing loss, CN dimensions and signal intensity.

Methods and Materials: Institutional review board approval was obtained.Study group consisted pre-operative MRI of the patients who underwent a CI surgery for bilateral profound postlingually sensorineural hearing loss between January 2013 and May 2018. The control group consisted of images of patients whose MRI were performed due to vertigo. There were 20 patients 40 ears (13 male and 7 female) in study group and 27 patients 54 ears (16 male and 11 female) in control group. 1.5 Tesla MRI and 3D FIESTA sequences on axial and sagittal-oblique views were used. Cochlear nerve length (CNL), the angel between Cochlear nerve and brain stem (CNA), Cochlear nerve circumference (CNC) Cochlear nerve surface area (CNSA), Cochlear nerve signal intensity (CNSI), Facial nerve calibre (FNC), Facial nerve surface area (FNSA), Facial nerve signal intensity (FNSI), and the ratio of Cochlear nerve intensity (SIC) /Fascial nerve intensity (SIF) was measured for all individuals and semi-automated quantitative assesments were performed.

Results: Demographic data was similar for both proups ($p>1.0$) and variables were homogenous. The duration of hearing loss was mean 20.75±12.42SD (0.5-45) years in study group. There was no statistically significant difference between two groups in CNL ($p=0,856$) and CNA ($p=0,535$). There was no intensity loss in study group when compared to control group($p=0,080$). However CNC and CNSA was significantly different ($p<0.001$). FNSI was statistically different in two groups ($p=0,006$). However Cochlear nerve intensity (SIC)/Fascial nerve intensity (SIF) ratio was similar in both groups ($p=0,363$). Although FNC was statistically different among two groups ($p=0,008$), FNSA was similar for both groups ($p=0,055$). Pearson correlation analyses revealed no correlation between the duration of the hearing loss, CNC, CNSA and intensity of the nerve.

Conclusion: There are limited studies investigating the same parameters for patients with postlingually hearing loss (PHL). In our study; CNC, CNSA and FNC are found to be significantly different in PHL patients when compared to control group and this is not correlated with the duration of hearing loss. Nerve fiber transition between CN and FN can be the reason of change of FNC diameter in PHL patients.

Disclosure: No significant relationships.
Keywords: cochlear nerve, Facial nerve, magnetic resonance imaging, profound hearing loss

### SOPS 2.5

### Screening for pineal trilateral retinoblastoma revisited: a meta-analysis

*M. De Jong[1], W. Kors[2], A. Moll[1], P. De Graaf[1], J. Castelijns[1], R. Jansen[1], B. Gallie[3], S. Soliman[1], F. Shaikh[1], H. Dimaras[3], T. Kivelä[4]; [1]Amsterdam/Netherlands, [2]Utrecht/Netherlands, [3]Toronto/Canada, [4]Helsinki/Finland*

Short Summary: In this meta-analysis we found that age of ocular retinoblastoma diagnosis is independent from age at pineoblastoma diagnosis and that retinoblastoma patients are at risk for pineoblastoma until the age of approximately 40 months at a constant rate during this period. Screening with MRI beyond the routine baseline brain scan at retinoblastoma diagnosis, when 50% of pineoblastomas are detected, would require at least an additional 311 scans to detect 1 pineoblastoma.

Purpose/Objectives: Up to 4% of patients with retinoblastoma are at risk for pineal trilateral retinoblastoma (pineoblastoma). Survival after pineoblastoma has improved considerably from virtually 0 to about 50%. Early detection and proper treatment is essential for survival. The current evidence is unclear on the usefulness of screening for pineoblastoma; in literature until the age of 5 years patients are assumed to be at risk. To determine whether screening might be useful and when, we performed a meta-analysis to determine until what age children are at risk for pineoblastoma, whether the onset of pineoblastoma is linked to the age at which eye tumor develops and the lead time between a detectable tumor and symptomatic pineoblastoma.

Methods and Materials: We searched PubMed (Medline) and Embase between January 1, 1966 and February 27, 2019 for literature that evaluated trilateral retinoblastoma. The Mann-Whitney U test (two-sided) was used to compare subgroups. Pearson's r was used to calculate a correlation between two continuous variables.

Results: In total 135 pineoblastoma patients were included in the analysis. Ninety percent of patients with asymptomatic pineoblastoma (20/21) were diagnosed before 40 months. Age at pineoblastoma diagnosis was independent of age at intraocular retinoblastoma diagnosis (testing for dependency: $P > 0.4$). The lead time between asymptomatic and symptomatic pineoblastoma was ~1 year. From this, we calculated that for a screening program with 1 MRI scan every 6 months after retinoblastoma diagnosis (~6 months of age) until the age of 3 years, at least 311 scans would be required to detect 1 pineoblastoma.

Conclusion: This study suggests that retinoblastoma patients are at risk for pineoblastoma within a narrower time- period than previously assumed and the age of retinoblastoma diagnosis has no influence on the age of pineoblastoma diagnosis. Screening would require numerous MRI scans to diagnose 1 pineoblastoma and even more to save 1 life.

Disclosure: No significant relationships.
Keywords: screening, trilateral retinoblastoma, retinoblastoma, meta-analysis, pineoblastoma, MRI

### SOPS 2.6

### Dynamic Fast Imaging Employing Steady-State Acquisition (FIESTA) MRI sequence of the vocal tract on overtone singer; our preliminary experience

*F. Barbiera[1], B. Murmura[2], A. Lo Casto[2], G. Bortoluzzi[3], I. Orefice[3], A. Trigona[4], G. Gucciardo[4]; [1]Sciacca/Italy, [2]Palermo/Italy, [3]Oristano/Italy, [4]Agrigento/Italy*

Short Summary: We perform dynamic MRI on overtone singer to demonstrate physiological changing of vocal tract' structures and, for extension, to demonstrate the feasibility of MR imaging to study normal articulation of speech and singing

Purpose/Objectives: To demonstrate physiological changing of vocal tract' structures during overtone singing with commercial MRI Fast Imaging Employng Steady State Acquisition (FIESTA) dynamic sequence .

Methods and Materials: We employed a General Electric Signa, HDxt 1.5 Tesla MRI scanner and a 16 channel head-and-neck coil as a receiver. Commercial FIESTA sequence for cardiac imaging was used, without cardiac gating. We acquired a single slice on mid-sagittal plane with the following parameters: TR 3,1 ms, TE 1,3 ms, bandwith 90,9 khz, 1 nex, Flip angle 15°, FOV 23, thickness 8 mm, 128x128 matrix. We reached a temporal resolution of 0,155 sec (7 image/sec) repeated for 512 times with an overall acquisition time of 1 minute and 19 seconds. These sequences were acquired while the singer performed a predetermined singing sequence. To cancel the noise overimposed, the dynamic MRI was subsequently dubbed in studio with the audio of the preset overtone sing. Dubbed MR images were analyzed with Overtone Analyzer® Software and different sound frequencies were identified and pointed out as colored lines

Results: For each overtone technique we evaluated the movement of lips, tongue, velopharingeal closure and distance between tongue and pharyngeal posterior wall/velum palatinum. In the first acquisition, using a technique named Ezengileer, we saw that the tip of the tongue, kept in touch with the hard palate, modulates the overtones of the fundamental pitch. Meanwhile, it is clear that a percussive sound is achieved by intermittent velopharyngeal closure. In the second acquisition, with a technique named Sygyt, we analyzed the manipulation of the entire range of overtones, from the lowest to the highest back and forth, noticing a progressive increase of the distance between lips and a shortening of the tongue. In the third acquisition the subject used another style called J-technique, where the tongue stays next to palate, velum palatinum and pharyngeal wall. The overtones are modulated by an intermittent movement of the medium part of the tongue, which moves away and gets closer to the palate.

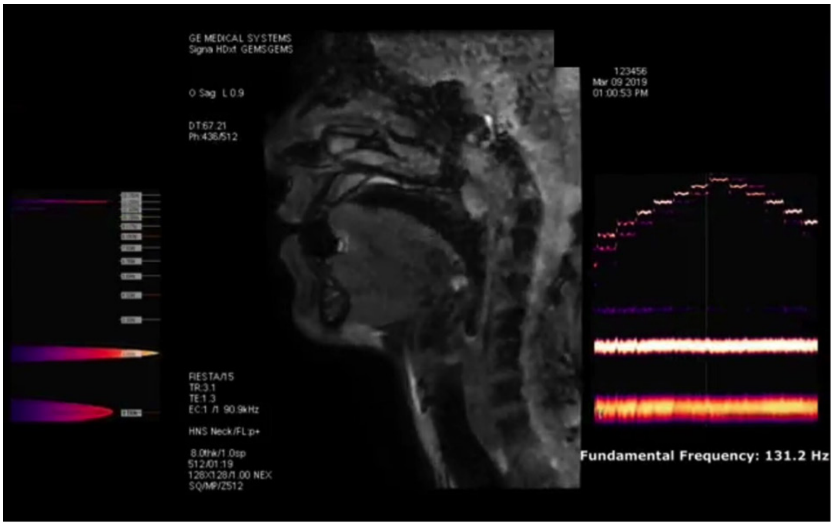

Conclusion: The preliminary data of our study demonstrate that FIESTA dynamic MRI sequence can be used to depict changing of position of vocal tract's structure in overtone singer techniques with excellent temporal and anatomic resolution.

Disclosure: No significant relationships.

Keywords: Vocal tract, FIESTA, Overtone singer, Dynamic MRI

### SOPS 2.7

### Skull base meningoencephalocele in patients with elevated intracranial pressure: imaging findings

*S. Parlak, A. Akgoz Karaosmanoglu, K. Karli Oguz; Ankara/Turkey*

Short Summary: Spontaneous cerebrospinal fluid (CSF) fistulas and meningoencephalocele(ME)s in the skull base can occur in presence of elevated intracranial pressure (EIP). CT and MRI findings of patients who had skull base MEs and secondary EIP were retrospectively examined. Temporal bone was the most common site of MEs with bilateral and multiple involvement. One patient had also otorhea due to CSF fistula in mastoid bone in addition to multiple MEs in cranial base. MEs in the skull base can be acquired in cases with EIP of various etiologies and radiologists should be aware of this situation.

Purpose/Objectives: Etiopathogenesis of spontaneous cerebrospinal fluid (CSF) fistulas and meningoencephalocele(ME)s in skull base is a multifactorial process. Elevated intracranial pressure (EIP) and anatomic structural weakness of skull base are two main causative reasons.

The association of spontaneous encephalocele with CSF leak and idiopathic intracranial hypertension is well documented in literature. However, there are few reported anectodal cases describing encephalocele formation in patients with known causes of raised intracranial pressure, such as tumor or hydrocephalus. We aimed to report radiologic findings of patients with skull base MEs secondary to known causes for EIP in our case series.
Methods and Materials: Computed Tomography (CT) and Magnetic Resonance Imaging (MRI) findings of several patients with secondary MEs and EIP were retrospectively examined.
Results: The study group consisted of 7 patients (F/M: 3/4) with a mean age of 28.2 years (range: 8/12-55 years). 4 patients were children (8/12-14 years). All patients but two (n=5; 71%) had imaging findings of had EIP at the time of imaging, such as optic nerve tortuosity, optic nerve sheath enlargement and enlargement of sella turcica. The patients suffered from EIP due to craniosynostosis (28%, n=2), hydrocephalus (57%, n=4). One patient had long term history of chronic venous sinus thrombosis although no sign of EIP on available single imaging study. Pediatric patients with hydrocephalus were on follow-up for Chiari type 1 malformation (n=1) and Neurofibromatosis type 1 (n=1). Temporal bone was the most common site of MEs with bilateral and multiple involvement in 5 patients. Two patients had single ME in temporal bone and cribriform plate respectively. One patient had also otorhea due to CSF fistula in the mastoid bone in addition to multiple MEs in the anterior and middle cranial base.
Conclusion: MEs located in the skull base can be an acquired abnormality due to several underlying reasons of EIP. Radiologists should be aware of this situation to manage patients appropriately.
Disclosure: No significant relationships.
Keywords: Encephalocele, skull base, elevated intracranial pressure

### SOPS 2.8

### Normal facial nerve enhancement on isotropic 3D fast spin echo T1 SPACE / CUBE sequences at 1.5T

*R. Warne[1,2], O. Carney[1], S. Connor[1]; [1]London/United Kingdom, [2]Perth/Australia*

Short Summary: Normal patterns of petrous bone facial nerve gadolinium enhancement must be established in order to distinguish from pathological processes, and these have been described in the context of spin echo and 3D gradient echo sequences. With increasing neuroimaging applications of 3D fast spin echo sequences such as T1 SPACE (Siemens) and CUBE (GE), we aimed to reappraise the normal variation of facial nerve gadolinium enhancement.
Purpose/Objectives: To establish normative data on 3D FSE CUBE and SPACE facial nerve gadolinium enhancement.
To assess the frequency of ‘fundal’ facial nerve enhancement on T1w 3D FSE sequences-an enhancement pattern which has previously been shown to be rare with standard spin echo sequences, and is associated with clinical presentations of Bell’s palsy.
Methods and Materials: A retrospective search of the radiology information system was performed (over 2 years) for patients undergoing a MRI protocol which included pre and post gadolinium 3D FSE T1w sequences with coverage of the petrous bones. A prior exclusion criteria (n=9) were any leptomeningeal or neural MRI abnormality, previous intracranial or petrous bone surgery, neurological signs, and any history of facial nerve or other cranial nerve palsy. 3D T1w SPACE (n=43) or CUBE protocols (n=21) (see table 1) were performed both pre / post contrast (without fat saturation) acquired immediately after intravenous bolus injection of 0.1mmol kg -1 Gd-DTPA The signal intensity was graded at each segment of the facial nerve on pre and post gadolinium imaging by two independent observers as 0, CSF intensity; 1, white matter intensity of brain stem parenchyma; 2, signal intensity between brainstem white matter and subcutaneous fat and; 3, intensity of subcutaneous fat. Mild enhancement increased by one grades and marked by two grades.
Results: 64 patients (21M, 43F; mean age 42 M 47 F, age range 24-85) and 128 facial nerves were reviewed.
The unenhanced signal intensity of the SPACE and CUBE six facial nerve segments were simlar (average signal intensity of 1).
Significantly higher contrast-enhanced SI was present in the SPACE / CUBE intracanalicular- fundal (15%), labyrinthine (1%), geniculate ganglion (96%), tympanic (39%) and mastoid segments (37%).
Conclusion: This current study is the first to systematically compare between enhancement characteristics of facial nerve using 3D T1w FSE techniques,
with identification of ‘fundal’ enhancement in these normative data sets in 15% of cases.
Disclosure: No significant relationships.
Keywords: Facial nerve, isotropic 3D fast spin echo T1 SPACE / CUBE sequences at 1.5T, Fast spin echo, Normal facial nerve enhancement, CUBE / SPACE 3D FSE

### SOPS 2.9

### Ultrasound occasional findings of thyroglossal duct anomalies in the root of the tongue

*A. Nadtochiy, O. Vozgoment; Moscow/Russian Federation*

Short Summary: Thyroglossal cysts and fistulas – congenital thyroglossal duct anomaly (TGDA) constitute significant part of maxillofacial pathology. TGDA clinical manifestations usually provoked by inflammation. The main method of TGDA treatment is surgery but the relapses occur in 5-40% of cases.
Purpose/Objectives: Evaluation the incidence of preclinical forms of TGDA in population and also to identify and systematize the “unconscious complaints” of patients associated with this pathology.

Methods and Materials: Ultrasound screening of tongue root (TR) was performed in 3681 patients (aged 2-73 years) with norelated maxillofacial and neck diseases. If asymptomatic TGDA revealing the purposeful study of the patient's feelings was applied to identify "unconscious complaints".
Results: Asymptomatic TGDA in the TR were revealed in 316 (8.6%) patients: hypoechoic line structures (HELS –fistulas or unreduced fibrous cords) –247 (6.7%/78.2%); cysts –44 (1.2%/13.9%); cysts in combination with fistulas –27 (0.7%/8.5%). Thus, asymptomatic cysts were detected in 71 patients: 1.9% of patients in population and 22.4% of patients with clinically asymptomatic TGDA. Iin 31 (43.7%) patients cysts had contact with hyoid bone, 35 (49.3%) –to the TR mucosa, in 5 (7%) – located in TR center. Most often "unconscious complaints" in patients with "asymptomatic" TR cysts: periodic appearance of mucus in the throat and its coughing –49 (69%) pts, periodic difficulties in swallowing and tongue articulation –27 (38%) patients. Variants of HELS localization were revealed: in 153 (61.9%) patients HELS had contact with hyoid bone, 82 (33.2%) – with TR mucosa, and 12 (4,9) - the localization was mixed; in 211 (85.4%) revealed single HELS, in 36 (14,6%) – multiple.
Conclusion: Ultrasound examination of the tongue root allowed to determine the population frequency of preclinical forms of TGDA. Careful study of patient's anamnesis allows to reveal "unconscious complaints" indicating the presence of "asymptomatic" thyroglossal cysts of the tongue root.
Disclosure: No significant relationships.
Keywords: Thyroglossal duct anomalies, Ultrasound examination of Root of Tongue, Cysts and fistulas of Root of Tongue, Asymptomatic thyroglossal cysts

### SOPS 2.10

### Follow-up of juvenile angiofibroma: may an early post-op MRI replace serial follow scanning?

*G. Palumbo, M. Ravanelli, A. Schreiber, D. Farina, R. Maroldi; Brescia/Italy*

Short Summary: Early post-op MR after resection of juvenile angiofibroma (JAF) for the detection of residual disease has been already recommended by some authors. Nevertheless, since this surveillance policy has never been validated, its findings do not influence the follow-up schedule. Based on our experience we propose that the early post-op MR may be a valuable prognostic tool in the setting of operated JAF and that follow-up schedule may be modified accordingly.
Purpose/Objectives: MR is the technique of choice for treatment planning and follow-up of juvenile angiofibroma (JAF). The optimal follow-up schedule is not universally accepted; according to some authors, serial MR follow up scans may be avoided if no residual disease is seen on an early post-op MR scan. Here we propose a personalized follow-up strategy based on the findings of early postoperative MR, performed no later than 72 hours after surgery (72hMR).
Methods and Materials: In this prospective study, 27 males with JAF (median age=15 years, range 11-24 years) were enrolled from 2007 to 2018. Each patient underwent: a) preoperative embolization, b) endoscopic resection c) 72hMR; d) MR follow-up of at least 6 months. MR follow-up and histology (in reoperated persistent JAF) were considered the standard of reference. JAF persistence was defined on MRI scans in the presence of an early hyperenhancing nodular lesion, in areas preoperatively affected by JAF. 72hMR findings were considered negative, positive or suspicious by an expert head-and-neck radiologist.
Results: 72hMR showed no residual disease in 19 cases, residual disease in 6 cases, and suspicious findings in 2 cases. All positive 72hMR diagnoses were confirmed during follow-up (4/6) or histology after early resurgery (2/6). No persistence was observed in the follow up of the 19 patients with negative 72hMR. In 2/2 patients with suspicious findings on 72hMR, no lesion was found during follow up. Sensitivity, specificity, PPV and NPV were 100%, 90.5%, 75% and 100%, respectively.
Conclusion: The high NPV suggests that when the 72hMR scan is negative, serial follow up scans may be avoided. Positive results may lead to early reoperation (unintentional persistence) or be used as early baseline for the monitoring of the rate of growth.
Disclosure: No significant relationships.
Keywords: Juvenile angiofibroma, MRI, follow-up, surgery

### SOPS 2.11

### Evaluation of different MRI protocols for orbital soft tissue segmentation

*R. Willaert[1], K. Orhan[1,2], J. Detobel[1], S. Bogaert[3], S. Ruiters[1], E. Shaheen[1], R. Jacobs[1]; [1]Leuven/Belgium, [2]Ankara/Turkey, [3]Ghent/Belgium*

Short Summary: Image post-processing and analysis is gaining importance in the field of orbital disorders. This study investigates the accuracy of soft tissue segmentation using different dedicated protocols for orbital MRI.
Purpose/Objectives: Quantitative evaluation of orbital soft tissue volume is useful for analyzing disease stages, treatment response and planning surgical treatment in various orbital disorders. Enhanced MRI methods and post-processing analysis could provide more accurate volume estimations. This study aims to define and evaluate the most accurate orbital MRI protocols for semi-automatic soft tissue volumetry.
Methods and Materials: A pilot study was performed to test 12 different orbital MRI acquisition protocols (3T, Siemens Magnetom, 64-channel head coil). After qualitative and quantitative analysis, three dedicated MRI sequences were selected for an in-depth evaluation. Three fresh cadaveric pig heads were first scanned with 3 MRI protocols, next a CT scan was performed, and orbital soft tissues were anatomically dissected at the end. Post-processing software programs (Synapse 3D and Mimics 21.0 innovation suite) were used to semi-automatically segment different orbital soft tissues (globe, extra-ocular muscles, orbital fat). Appropriate statistical analysis was done to compare results between different MRI protocols and between MRI, CT and anatomical dissection (gold standard). Intra-observer variability was assessed.
Results: Semi-automatic 3D volumetric measurement of orbital fat was most accurate with a TSE-DIXON based protocol. For the extra-ocular muscles and globe volume a T2-Space protocol was most reliable. The soft tissue volumetry on MRI is sufficiently reliable when comparing measurement errors to CT and anatomical dissection. Segmentation of muscle tissue is most accurate, results of fat volumetry showed the largest error and intra-observer variability.
Conclusion: Accurate volumetric analysis of orbital soft tissues is feasible and reliable when using different dedicated MRI protocols.
Disclosure: No significant relationships.
Keywords: CT, orbital soft tissue, post-processing, Segmentation, volumetric analysis, MRI

### SOPS 2.12

### Venous malformations of the head and neck: presentation 18 cases and review of the literature

*A. Modoni, M. Magli, F. Franco, T. Magli; Martina Franca/Italy*

Short Summary: Background: Venous malformations (VMs) are a common type of vascular malformation. However, their causes and management remain unclear, and few studies specific to VMs of the head and neck have been reported. This study describes our experiences with VMs of the head and neck
Purpose/Objectives: Early and accurate diagnosis and appropriate treatment according to individual symptoms are important for successful treatment of VMs. If treatment is delayed, the lesions can worsen, or recurrence becomes more likely. Therefore, VMs require a multidisciplinary approach for early and accurate diagnosis.
Methods and Materials: Methods the 18 Patients were studied by head and neck MR with a diagnosis of MVS in the 2018-2019 period
Results: The suspected diagnosis of MVs was based on the results of MRI or biopsy. Furthermore, MVs have been studied using ecocolor doppler and CT. VMs were more common female patients 62 %, and 38 % of patients developed symptoms at the age of ten or younger. The main sides involved were the cheek, 26 %, and lip area 24 %.cheek Small lesions less than 5 cm in diameter accounted for 58 % of cases, and well-defined types were slightly more prevalent at 54 %. Improvement was observed in 70 % of treated patients.
Conclusion: Successful treatment of VMs. depends for early and accurate diagnosi made with imaging. The imaging with clinical aspects are both very important for successful treatment of VMs. VMs require a multidisciplinary approach for early and accurate diagnosis.
Disclosure: No significant relationships.
Keywords: VMs, imaging, early diagnosis

### SOPS 2.13

### Should postlaminar optic nerve tumor invasion into the outer layers be considered a risk-factor for leptomeningeal spread of retinoblastoma?

*R. Jansen, M. De Jong, P. Van Der Valk, F. Abbink, M. Bosscha, J. Castelijns, A. Moll, P. De Graaf; Amsterdam/Netherlands*

Short Summary: We present a 6 year old boy with unilateral retinoblastoma of the left eye with postlaminar optic nerve tumor invasion into the outer layers on MRI and histopathology. Unfortunately 5 months later the patient developed leptomeningeal metastases despite systemic chemotherapy. In this study we discuss whether retinoblastoma invasion into the outer layers of the optic nerve should be considered a risk-factor for leptomeningeal spread of retinoblastoma.
Purpose/Objectives: Is optic nerve invasion into the outer layers up to the pia mater of the postlaminar optic nerve a risk factor for developing metastases in retinoblastoma? In this paper we present a patient with limited postlaminar optic nerve invasion that developed cerebrospinal fluid metastases.
Methods and Materials: This study comprised a case report of a boy aged 6 years who was clinically diagnosed with sporadic unilateral retinoblastoma, subsequent MRI was performed. After enucleation the eye was assessed histopathologically.
Results: MRI showed that the tumor had a maximum diameter of about 20 mm with a (near) total retinal detachment (figure 1, pre- and postcontrast T1-images [a and b] and subtraction of a and b [c] showing postlaminar invasion). Almost the entire retina was invaded by tumor cells. The distal part of the postlaminar optic nerve showed contrast enhancement and the distal part of the nerve appeared slightly thickened up to 3.2 mm, versus 2.7 mm of the contralateral normal right optic nerve (figure 1d-e, FIESTA images showing the distal optic of affected and normal eye). It was decided to enucleate the eye. The enucleated specimen included 10 mms of optic nerve with 3 mm of postlaminar invasion (i.e., the resection margin was free). Therefore, it was decided that no further diagnostics were warranted at that time, such as evaluating cerebrospinal fluid for malignant cells. MR images correlated with the findings on histopathology: postlaminar invasion with tumor reaching the outer layers of the optic nerve up to the pia mater and thickening of the distal optic nerve, see figure 2. Following protocol this patient received six courses of adjuvant systemic chemotherapy. Unfortunately, after five months this patient returned with leptomeningeal spread of the tumor and died quickly thereafter.

*Figure 1*

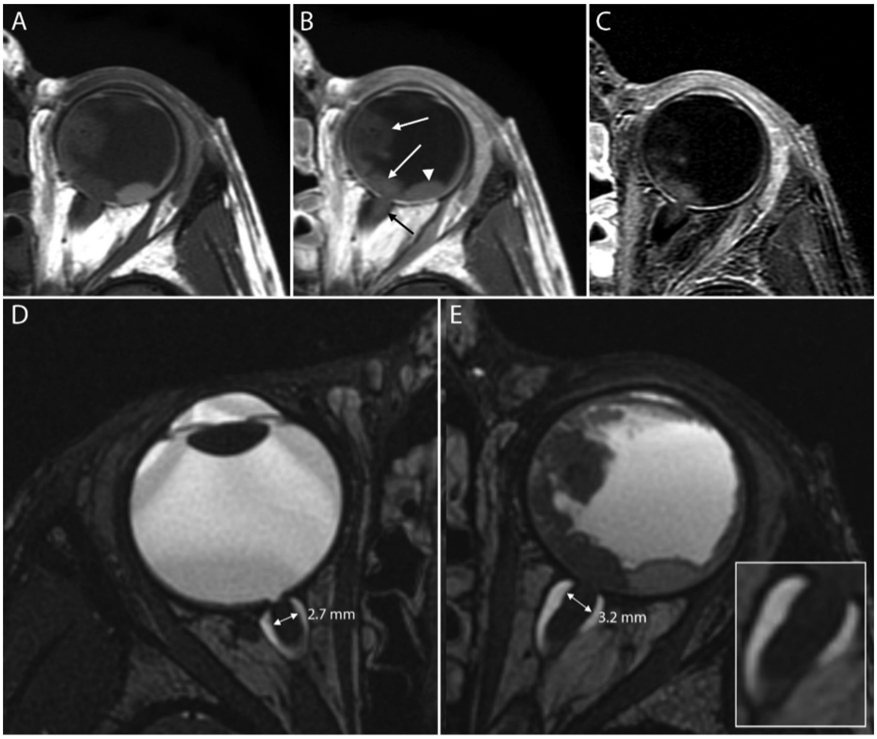

*Figure 2*

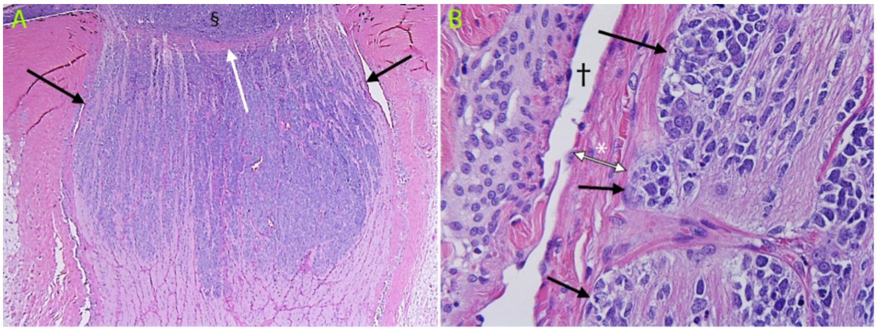

Conclusion: A resection margin with tumor cells is recognized as a risk factor for metastasis, but perhaps the proximity of tumor cells to the leptomeninges should also be judged increased risk for metastatic spread.

Disclosure: No significant relationships.

Keywords: retinoblastoma, MRI, Postlaminar invasion, Metastatic risk factor, Leptomeningeal metastasi

### SCIENTIFIC ORAL PRESENTATION SESSION: FACIAL

### SOPS 3.1

### Evaluation of the relationship between degenerative changes and bone quality of mandibular condyle and articular eminens in temporomandibular joint dysfunction by CBCT

*F. Namdar Pekiner, G. Ulay; Istanbul/Turkey*

Short Summary:
Objectives: The purpose of this study is to evaluate the correlation between degenerative bone changes and the bone quality of the mandibular condyle and articular eminence in patients with temporomandibular joint dysfunction with cone beam computed tomography (CBCT).
Methods and Materials: The study group consisted of 100 patients (83 female and 17 male) with TMD. Cases with clinical diagnosis of TMD were evaluated with CBCT. In the radiological examination, degenerative bone changes in condyle and articular eminence were determined and bone quality was classified according to BQI scale and their relationship was examined.
Results: The majority of cases with temporomandibular joint dysfunction were women. When the distribution of TMD was observed in the age groups, it was found 65 % in the 20-40 age range. The most common degenerative bone changes in mandibular condyle are flattening and erosion. The most common grade of bone quality was found Type III in condyle and articular eminence of patients with degenerative joint disease.
Conclusion: CBCT is a convenient radiological imaging modality for evaluating the bone quality and degenerative bone changes of TMJ.
Disclosure: No significant relationships.
Keywords: TMJ, BQI, cone-beam computed tomography, degenerative change, temporomandibular joint dysfunction

### SOPS 3.2

### Nasal CT anatomy of different ethnic configurations and aesthetic deformities

*M. Dutova, D. Lezhnev, D. Davydov; Moscow/Russian Federation*

Short Summary: MSCT permits to evaluate the different types of nasal deformities and ethnic specialties and to identify their anatomical base. There is a correlation between the external configurations and variants of pyriform apertures (P=0.722) and nasal bones (P=0.562) ($p<0,05$).
Purpose/Objectives: The aim: to identify the anatomical variants of nasal bones and pyriform apertures in view of normal ethnic configuration of external nose and different types of nasal deformities.
Methods and Materials: 132 patients with aesthetic nasal deformities seeking rhinoplasty underwent multislice computed tomography (MSCT, 64-slice, Philips, USA) with the image processing. Also we included the groups of patients with normal middle-European (n=43), Balkano-caucasian (n=33) and Mongoloid (n=24) nasal configurations who performed MSCT by other indications (inflammatory diseases and cysts of maxillofacial area).
Results: A total of nasal deformities were divided on the groups: rhinokyphosis (n=37), long nose (n=16), combined deformity like a hidden hump (n=46), short nose (n=16), wide nose (n=17). The most frequent variants of pyriform apertures in all patients groups were heart (n=84, 36.2 %), drop (n=55, 23.3 %) and pear (n=34, 14.7 %) types. The persons with wide nose had the round (31.3%) and trapezoid forms (56.3%), the patients with long nose – rhomboid variant (25.0%). The most common variants of nasal bones in all groups were 2$^{nd}$ (n=93, 40.1%), 5$^{th}$ (n=51, 22.0%), 6$^{th}$ (n=31, 13.4%), 7$^{th}$ (n=29, 12.5%) types according to Lang and Baumeister. The caudal part of nasal bones was also estimated due to variability of different marginal defects (symmetrical/asymmetrical, deep, unique) in the overwhelming amount of patients (78.4%). The generality of Asians had the heart variant (87.5%) and the 7$^{th}$ (45.8%) type with smooth, symmetrical lacunae in 75.0% patients or a whole nasal edge (20.8%). One third of Balkano-Caucasian persons had the 4$^{th}$ and 8$^{th}$ types. There was a correlation between the facts of deformities or ethnic specialties and variants of pyriform apertures (P=0.722) and nasal bones (P=0.562) was obtained ($p<0.05$).
Conclusion: MSCT permits to evaluate the different types of nasal deformities and to identify their anatomical base. The dominant variants of pyriform apertures are the drop, heart and pear types as well as 2$^{nd}$, 5$^{th}$, 6$^{th}$, 7$^{th}$ forms of nasal bones. Every kind of deformities and ethnic specialties was described with their characteristic features of pyriform apertures and nasal bones with statistically significant relationships. These data should be used on the preoperative surgical stage for improving the outcomes of rhinoplasty.
Disclosure: No significant relationships.
Keywords: rhinoplasty, computed tomography, nasal bone, pyriform aperture, nose, nasal deformity

### SOPS 3.3

### Retrospective morphologic MRI evaluation of lateral pterygoid muscle in patients with referred symptoms of temporomandibular joint disorders

*F.P. Lombardo[1], T. Beale[2], G. Lo Meo[1], R. Bignone[1], E. Bruno[1], A. Lo Casto[1]; [1]Palermo/Italy, [2]London/United Kingdom*

Short Summary: The lateral pterygoid muscle (LPM), and particularly his superior belly (SHLP), has been postulated to undergo spasm and be involved in internal derangement of the temporomandibular joint (TMJ). We performed a retrospective evaluation of 78 TMJs by measuring signal intensity and thickness within the LPM and correlated those findings with the the presence of disk displacement and osteoarthritic changes of TMJ. A statistically significant increase of relative SHLP thickness has been found in patients with internal disk derangement; furthermore, a statistically significant correlation has been found between internal derangement and non-reductability of disk displacement; osteoarthritic changes seem to be a cofactor in the genesis of the spasm.

Purpose/Objectives: The goal of this retrospective study was to determine the morphologic changes of LPM in patients complaining symptoms of temporomandibular disorders by measuring signal intensity and thickness within the superior and the inferior (IHLP) heads of the LPM.

Methods and Materials: A retrospective review of 39 MR TMJ evaluations was performed in Policlinico Universitario “Paolo Giaccone” (Palermo, Italy) and in University College Hospital (London, United Kingdom) from January 2018 to March 2019. A total of 78 TMJs was found. We measured signal intensity in SHLP and IHLP using representative regions of interest placed in the image that represented the midportion of the muscle bellies, obtaining a Signal Ratio (SR). We measured thickness in the midportion of SHLPM and IHLPM in the craniocaudal dimension, obtaining a Thickness Ratio (TR). We used a 2-tailed Student t test to compare SR and TR between several subgroups of patients; we used Chi-square test to evaluate the relationship between severity and direction of disk dislocation.

Results: Comparison of TR demonstrated significant increase in SHLP thickness between: anteromedial dislocation and lateral dislocation; anteromedial dislocation and no dislocation; non-reductible disk dislocation and reductible disk dislocation; patients with osteoarthritic signs and without osteoarthritic signs; patients with no disc dislocation and osteoarthritic signs and with both no disc dislocation nor osteoarthritic changes.

Comparison of SR demonstrated no significant correlation between subgroups. Comparison between direction and severity of disc derangement demonstrated significant strong correlation between anteromedial dislocation and non-reductibility of dislocation.

*SHLPM/IHLPM thickness ratio*

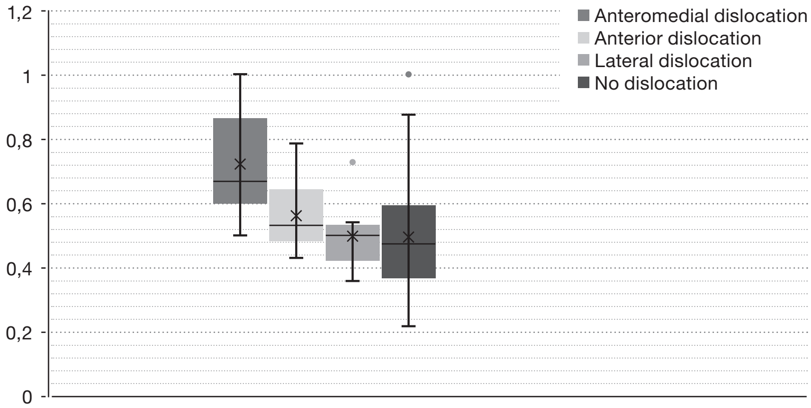

Conclusion: The increase of the thickness of SHLP seems related to internal derangement of the disk; internal derangement itself seems significantly related to a condition of non-reductibility of disk dislocation, with prognostic worsening of the temporomandibular disorder. Osteoarthritic changes seem to be a feasible cofactor in the genesis of the spasm, even in patients without disk dislocation.

Disclosure: No significant relationships.
Keywords: TMJ, spasm, lateral pterygoid muscle, MRI

### SOPS 3.4

### Sinonasal IgG4-related disease: role of CT/MRI and emerging imaging patterns

*A. Dragan, R. Lingam; Harrow/United Kingdom*

Short Summary: IgG4-related disease is a newly defined concept, representing a group of conditions which share similar clinical, serological and histological characteristic. The underlying pathophysiology is yet uncertain. The condition was first described in the pancreas, but in recent years more and more sites of disease have been reported. In 2012 an international consensus set out clear diagnostic criteria, independent of the body site affected by the disease.

Purpose/Objectives: In the Head and Neck region, the salivary glands or the orbits are already well recognised and described sites of disease. In contrast, IgG4-related disease of the nose and paranasal sinuses has been much more sporadically reported in the literature, mainly as independent case reports.

The marked and rapid response to steroid treatment is one of the main features, underlining the importance of a rapid diagnosis. However, the main differential diagnoses of this condition in the sinonasal region are invasive fungal sinusitis (where steroid administration could lead to patient demise) or cancer. Hence a correct prompt diagnosis is essential for these patients.
Methods and Materials: We present here a case series of IgG4-related disease of the nose and paranasal sinuses, with special emphasis on the imaging features of the disease.
Results: We describe each case including clinical presentation, imaging modalities employed for diagnosis, also reviewing the serological and histopathological findings, treatment used and the subsequent outcomes. With each case, we will identify and reinforce an emerging pattern for the imaging findings, on multiple modalities, on both morphological and functional imaging. Identifying this pattern will allow the reporting radiologist, working in a multidisciplinary setting, to suggest IgG4-related disease as a differential diagnosis for the appropriate cases. Raising the suspicion for this condition will enable the clinician and the histopathologist to run specific serological or immunohistological tests which serve as diagnostic criteria.
Conclusion: IgG4-related disease is a new concept in medicine, but its diagnostic criteria have been clearly defined. The treatment is markedly different from that of its main differential diagnoses (cancer, but even more importantly invasive fungal sinusitis), which makes the quick and correct diagnosis essential. The reporting radiologist has the great opportunity of raising suspicion of the condition early, by recognising the imaging pattern of disease, thus enabling clinicians and pathologists to proceed to specific diagnostic tests, which will allow a prompt and accurate diagnosis.
Disclosure: No significant relationships.
Keywords: IgG4 related disease, sinonasal tract, CT, MR

### SOPS 3.5

**Dental disease incidentally iscovered in head and neck CT Studies**

*S.S. Messina, P. Purpura, G. La Tona, F.P. Lombardo, R. Bignone, A. Lo Casto; Palermo/Italy*

Short Summary: Oral health, according to WHO, is a key indicator of overall health, wellbeing and quality of life. Although in most cases dental disease is not “life threatening”, however it can lead to serious and sometimes even fatal complications. Multidetector CT (MDCT) is a widespread imaging technique that provides information on head and neck disease. Dental disease can be recognized at routine CT of the head and neck, but it is often overlooked due to the lack of knowledge by general radiologists.
Purpose/Objectives: The aim of this study is to report the prevalence of dental disease incidentally detected on head and neck CT exams reported by a head and neck radiologist.
Methods and Materials: A retrospective analysis was conducted on Radiology Department RIS-PACS from January 2017 to April 2019. 816 patients underwent routine head and neck MDCT performed for non-dental indications, reported by a head and neck radiologist who indicated incidental dental disease as: carious lesions, dental developmental anomalies, periapical lesions, periodontal/bone diseases, residual tooth roots, dental developmental anomalies, dental fractures and odontogenic orofacial infections. 578 brain (70%), 163 paranasal sinus (20%), and 75 (10%) neck MDCT studies were reviewed.
Results: 248/815 (30%) patients showed dental disease (avarage age was 53.5 years old, between 16 and 92 years old): carious lesions were found in 157/816 (29%), periodontal/bone disease in 111/815 (21%), periapical lesions in 105/815 (20%), residual tooth root in 103/815 (19%), dental developmental anomalies in 53/815 (10%), tooth trauma in 5/815 (1%), and odontogenic orofacial infection in 4/815 (1%) patients.
Conclusion: A high prevalence of dental disease was incidentally detected on routine head and neck CT exams by a head and neck radiologist. General radiologists should be aware that reporting incidental dental disease could not only prevent tooth loss and even fatal complications related, but dental disease increases the risk of many systemic ones, e.g. cardiovascular, respiratory, diabetes, rheumatoid arthritis.
Disclosure: No significant relationships.
Keywords: Dental Desease, Odontogenic orafacial infection, Carious lesions

### SOPS 3.6

**Ultrasonographic assessment of the capsular width in the temporomandibular joint effusion**

*L.M. Lenghel[1], N. Bolog[2], S. Buduru[1], D. Leucuta[1], M. Baciut[1], G. Baciut[1], H. Rotar[1], C. Dinu[1], D. Talmaceanu[1]; [1]Cluj Napoca/Romania, [2]Reinach/Switzerland*

Short Summary: High-resolution ultrasonography (US) is not used routinely for the TMJ pathology. The techinque is limited for the assessment of the disc due to the presence of the bony structures and it is unable to visualize the medial part of the condyle. In this study, US examination aimed to identify the presence of joint effusion by measuring the capsular distention between the superior-lateral surface of the mandibular condyle and the bone surface of the articular eminence. The capsular width can be used as an indirect diagnostic sign of TMJ effusion and synovitis.
Purpose/Objectives: The aim of the study was to evaluate the association between the increased capsular width of the temporomandibular joint (TMJ), assessed with high-resolution ultrasonography (US), and the joint effusion, assessed with MRI. Another objective was to establish the best cut-off value of the capsular width, which can be considered an indirect sign of TMJ synovitis and effusion.

Methods and Materials: 102 patients (204 TMJs) with signs and symptoms of temporomandibular disorders according to Research Diagnostic Criteria for Temporomandibular Disorders (RDC/TMD) were included in a prospective study. The US evaluation was performed on a machine with an 8–40 MHz linear transducer operating at 20 MHz. MRI examinations were performed using 1.5 T MRI equipment. US examination aimed to identify the presence of the joint effusion by measuring the capsular width between the superior-lateral surface of the mandibular condyle and the bone surface of the articular eminence. The ROC curve was analyzed to detect the optimal cut-off value for the capsular distention, which can be used as an indirect diagnostic sign of TMJ effusion and synovitis.
Results: The capsular width values measured on ultrasound were between 0.7-3.6mm. The best cut-off value was 2.05 mm with a sensibility of 55.9% and a specificity of 94.7% ($p<0.05$). The next optimal cut-off value was 1.75 mm with a sensibility of 82.4% and a specificity of 67.6%. The area under the curve was 0.78 (95 % CI 0.68-0.87).
Conclusion: Capsular width can be used as an indirect sign of TMJ effusion. High-resolution ultrasonography has not reached the diagnostic value of MRI in assessing TMJ inflammatory changes. However, this technique showed a good diagnostic accuracy and can be used in case of suspicion of TMJ synovitis and effusion.
Disclosure: No significant relationships.
Keywords: ultrasonography, Temporomandibular joint, magnetic resonance imaging

### SOPS 3.7

### In-vivo accuracy of tooth surface reconstruction based on CBCT and MRI

*T. Hilgenfeld, A. Juerchott, U. Deisenhofer, D. Weber, S. Rues, P. Rammelsberg, S. Heiland, M. Bendszus, S. Schwindling; Heidelberg/Germany*

Short Summary: Guided implant surgery (GIS) is a standard procedure in oral- and maxillofacial surgery. The current GIS workflow requires the alignment of virtual models (surface scans, e.g., from intraoral scanning) to 3D reconstructions of imaging. The *in-vivo* accuracy of CBCT-derived tooth surfaces in representative patients with dental restorations is unknown. Furthermore, we have developed a new method for dMRI-based tooth-surface imaging and directly evaluated its accuracy compared with that of CBCT. We hypothesized that dMRI could be used for surface reconstruction of non-restored teeth among patients in need of dental implants, which is a prerequisite for dMRI-based GIS.
This study revealed CBCT-derived occlusal tooth surfaces among implant patients to be highly accurate, even in the presence of multiple dental restorations. Furthermore, a new method for reliable and accurate 3D tooth-surface reconstruction based on dMRI was established. Geometric deviations for dMRI remained within the predefined equivalence margin, however, were still substantially lower than that of CBCT.

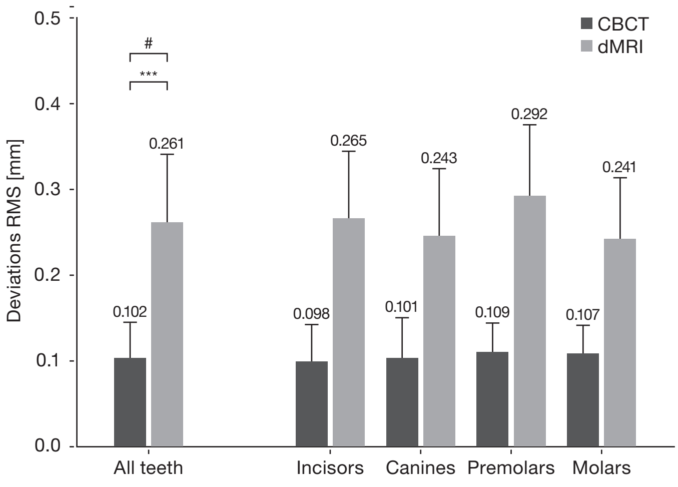

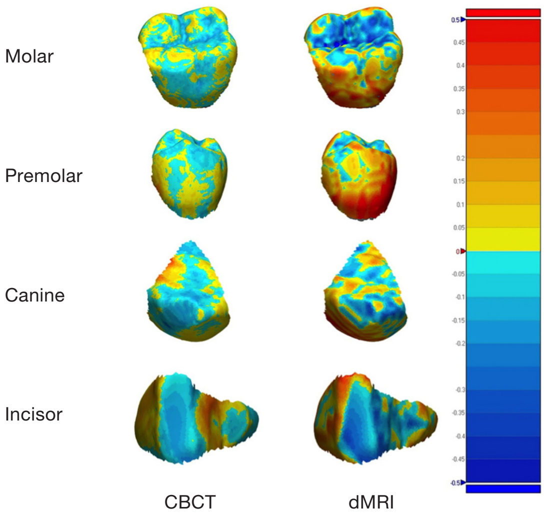

Purpose/Objectives: To determine tooth surface accuracy for guided implant surgery (GIS) in vivo in the presence of metallic artifacts using cone-beam computed tomography (CBCT) and dental magnetic-resonance-imaging (dMRI).
Methods and Materials: CBCT and 3 Tesla dMRI were prospectively performed in 22 consecutive patients (mean age: 54.4±15.2 years; mean number of restorations per jaw: 6.7±2.7). For dMRI a new method was introduced to visualize tooth surfaces using a splint and tooth paste. Altogether 92 teeth were included (31 incisor, 29 canines, 20 premolars and 12 molars). Surfaces were reconstructed semi-automatically and registered to a reference standard (3D-scans of stone models made from full-arch polyether impressions). Reliability of both methods was assessed using intraclass correlation coefficients. Accuracy was evaluated using the two one-sided tests procedure with a predefined equivalence margin of ±0.2 mm root mean square (RMS).
Results: Inter and intra-rater reliability of tooth surface reconstruction were comparable for CBCT and dMRI. Geometric deviations were 0.102±0.042 mm for CBCT and 0.261±0.08 mm for dMRI. For a predefined equivalence margin CBCT and dMRI were statistically equivalent. CBCT, however, was significantly more accurate ($p \leq 0.0001$). For both imaging techniques, accuracy did not differ substantially between different tooth types.

Conclusion: CBCT is an accurate and reliable imaging technique for surfaces of restoration-free teeth in vivo, even in the presence of metal artifacts. In comparison, the in-vivo accuracy of dMRI is lower, but the technique can still image tooth surfaces in satisfactory detail and within acceptable acquisition times for GIS.
Disclosure: No significant relationships.
Keywords: magnetic resonance imaging, Dental Impression Technique, Dental Implants, cone-beam computed tomography

### SOPS 3.8

### Dental age estimation with cone beam computed tomography

*H. Boyacioglu, N. Avcu, G. Akcıcek, N. Akkaya, S. Dural; Ankara/Turkey*

Short Summary: Radiological age determination methods in adult individuals are based on two-dimensional length and area measurements of the size of the pulp, which decreases with secondary dentin accumulation. With the use of cone-beam computed tomography (CBCT) images, pulp can be studied as three-dimensional volume. In the literature, the relationship between age and the pulp volumes for the lower and upper anterior teeth and lower premolar teeth was investigated. However, it was not found that the volume of secondary dentin accumulation was measured in the lower molar teeth.
Purpose/Objectives: The aim of this study was to measure the pulp volumes of the lower first molar and second molar teeth and to evaluate the correlation between the volume values and chronological age.
Methods and Materials: Total of 64 individuals CBCT images were selected. One oral radiologist performed the measurements of the pulp volumes. Date of birth was considered to obtain chronological age. The correlation between estimated and chronological ages was assessed using the Pearson correlation coefficient. Intraobserver agreement was investigated using interclass correlation coefficient. Statistical significance was set at $p<0.05$.
Results: The results show that there was a positive correlationship between estimated and chronological ages ($p<0.05$). There were significant intraobserver agreements in measurements.
Conclusion: The results herein imply the potential use of tooth volume measurements for forensic age estimation.
Disclosure: No significant relationships.
Keywords: cone beam computed tomography, dental age estimation, secondary dentine deposition

### SOPS 3.9

### Evaluation of osteoarthritic changes in temporomandibular joint and correlations with age: a retrospective CBCT study

*N. Koç; Ankara/Turkey*

Short Summary: Radiographic evaluation of degenerative changes in temporomandibular joint (TMJ) helps to monitor severity of the disease and therefore is essential even in absence of clinical symptoms. Cone beam computed tomography (CBCT) provides detailed visualization of the bony structures of TMJ.
Purpose/Objectives: To determine the prevalence of various bony changes of TMJ in patients with a wide range of age and to evaluate and correlate CBCT findings of TMJ osteoarthritis with age.
Methods and Materials: A retrospective study was implemented. CBCT images of 150 patients (43 males and 107 females) who were referred to Dentomaxillofacial Radiology Clinic for evaluation of TMJs were included in the study. Each TMJ was evaluated separately for the presence of any osseous changes in the condylar head and articular fossa/eminence and for the presence of joint space narrowing. The independent samples t-test was used to compare mean ages. The proportions of bone changes in the right and left sides were compared with the McNemar's test.
Results: The mean age of the sample was 37.26 years (range 10-90 years). A total of 101 (67.3 %) patients presented one or more osseous changes. The condylar changes were more common than the changes in the articular fossa/eminence. No significant differences were found between the right and left TMJs ($P=0.442$) , and between males and females for the presence of osseous changes ($P=0.335$). Statistically significant differences were found in the mean age between the absence and presence of condylar erosion ($P=0.001$), osteophytes ($P=0.001$), loose bodies ($P=0.005$), erosion in articular fossa ($P=0.006$), and between a normal and reduced joint space ($P=0.017$).
Conclusion: Degenerative changes of TMJ may reflect an age-related bone remodelling process or a physiologic adaptation. Older patients may have found to have more common degenerative changes such as erosion of the condyle and articular fossa, joint space narrowing, osteophytes and loose joint bodies.
Disclosure: No significant relationships.
Keywords: osteoarthritis, cone beam computed tomography, temporomandibular joint diseases

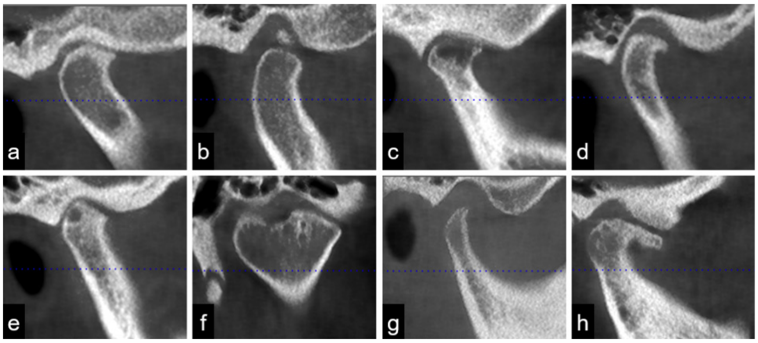

SOPS 3.10

### Assessment of juxta-apical radiolucency with cone beam CT

*S. Coskun, H. Boyacioglu, S. Dural; Ankara/Turkey*

Short Summary: The juxta-apical radiolucency (JAR), is a radiographic sign adjacent to the roots of mandibular third molars. It is a variation of the normal aspect of the trabecular bone in this region and pointed out as an important predictor of inferior alveolar nerve damage during third molar surgery.

Purpose/Objectives: This study aimed to investigate the association of JAR with third molar status and mandibular canal and also describe its radiologic features through cone beam computed tomography (CBCT).

Methods and Materials: CBCT images of 34 individuals (53 mandibular third molars) were evaluated for the presence of JAR. Than, the CBCT images were analysed to evaluate the position of the JAR and its relationship to the mandibular canal and buccal-lingual cortical plates. Descriptive statistical analyses were used.

Results: JAR was identified in 13 individuals and 24 mandibular third molars (10 right side, 14 left side). 58.3 % were on the apical third of the root and the rest was located along the mesial or distal surfaces of root. Close proximity to the buccal cortical plate was seen in 50 % of JAR located apically, whereas it was seen only 10 % of JAR located along the root surface. Only 20.8 % were associated with the mandibular canal.

Conclusion: The present study gives an insight into the relationship of JAR with mandibular canal and cortical plates using CBCT. However, future studies are warranted that use a larger sample size to validate the above findings.

Disclosure: No significant relationships.

Keywords: cone beam computed tomography, mandibular third molar, nerve damage

A
Abbink F. SOPS 2.13
Ai Q. SOPS 1.3
Akcıcek G. SOPS 3.8
Akgoz Karaosmanoglu A. SOPS 2.7
Akkaya N. SOPS 3.8
Anjari M. SOPS 1.2
Atalay B. SOPS 2.4
Attanasio M. SOPS 1.10
Avcu N. SOPS 3.8

B
Baciut M. SOPS 3.6
Baciut G. SOPS 3.6
Barabino E. SOPS 1.6
Barbiera F. SOPS 2.6
Barnsley H. SOPS 2.2
Beale T. SOPS 3.3
Bendszus M. SOPS 3.7
Bier G. SOPS 2.1
Bignone R. SOPS 3.3, SOPS 3.5
Bisdas S. SOPS 1.5
Bogaert S. SOPS 2.11
Bolog N. SOPS 3.6
Bongers M. SOPS 2.1
Bortoluzzi G. SOPS 2.6
Bosscha M. SOPS 3.1
Boyacioglu H. SOPS 3.8, SOPS 3.10
Braun K. SOPS 2.1
Brunetti A. SOPS 1.9
Bruno E. SOPS 3.3
Buccimazza G. SOPS 1.15
Buduru S. SOPS 3.6
Burd C. SOPS 1.2

C
Calistri L. SOPS 1.14
Campisi G. SOPS 1.10, SOPS 11.11
Caprioli S. SOPS 1.6
Carney O. SOPS 2.8
Carrafiello G. SOPS 1.15
Casella S. SOPS 1.6
Castelijns J. SOPS 2.5, SOPS 2.13
Chen W. SOPS 1.3
Cho S. J. SOPS 1.4
Cittadini G. SOPS 1.6
Cocozza S. SOPS 1.9
Colagrande S. SOPS 1.14
Connor S. SOPS 2.2 SOPS 2.2, SOPS 2.8
Cordova A. SOPS 1.10, SOPS 1.11
Coskun S. SOPS 3.10
Costantino C. 1.11
Cuocolo R. 1.9

D
Davydov D. SOPS 3.2
De Graaf P. SOPS 2.5, SOPS 2.13
De Jong M. SOPS 2.4, SOPS 2.13
Deisenhofer U. SOPS 3.7
Detobel J. SOPS 2.11
Dimaras H. SOPS 2.5
Dinu C. SOPS 3.6
Dragan A. SOPS 3.4
Dural S. SOPS 3.8, SOPS 3.10
Dutova M. SOPS 3.2

E
Elefante A. SOPS 1.9
Ernemann U. SOPS 2.1
Eser M. B. SOPS 2.4

F
Farina D. SOPS 2.10
Farneti A. SOPS 1.1
Feo N. SOPS 1.10, SOPS 1.11
Franceschelli G. SOPS 1.15
Franco F. SOPS 1.12, SOPS 2.12
Fusco R. SOPS 1.15
Fusco M. SOPS 1.15

G
Gallie B. SOPS 2.5
Gangemi E. SOPS 1.1
Garbajs M. SOPS 1.5
Genova V. G. SOPS 1.10
Goh V. SOPS 1.2
Gucciardo G. SOPS 2.6
Guha A. SOPS 1.2

H
Heiland S. SOPS 3.7
Hempel J. M. SOPS 2.1
Heztink F. SOPS 2.3
Hilgenfeld T. SOPS 3.7
Huinck W. SOPS 2.3

J
Jacobs R. SOPS 2.11
Jansen R. SOPS 2.5, SOPS 2.13
Joo L. H. SOPS 1.4
Juerchott J. SOPS 3.7

K
Kalcioğlu M. T. SOPS 2.4
Karli Oguz K. SOPS 2.7
Kim H. J. SOPS 1.4
King A. D. SOPS 1.3
Kivelä T. SOPS 2.5
Koç N. SOPS 3.9
Kors W. SOPS 2.5
Kostenko E. SOPS 1.16

L
La Tona G. SOPS 3.5
Lam W. J. SOPS 1.3
Lee J. H. SOPS 1.4
Lee M. K SOPS 1.4
Lei M. SOPS 1.2
Lenghel L. M. SOPS 3.6
Leucuta D. SOPS 3.6
Lezhnev D. SOPS 3.2
Lingam R. SOPS 3.4
Lo Casto A. SOPS 1.10, SOPS 1.11, SOPS 2.6, SOPS 3.3, SOPS 3.5
Lo Meo G. SOPS 3.3
Lombardo F. P. SOPS 3.3, SOPS 3.5
Lucev F. SOPS 2.3

M
Magli M. SOPS 1.12, SOPS 2.12
Magli T. SOPS 1.12, SOPS 2.12
Maroldi R. SOPS 2.10
Martens R. SOPS 1.8
Marzi S. SOPS 1.1
Matta D. SOPS 1.10, SOPS 1.11
Mens L. SOPS 2.3
Messina S. S. SOPS 3.5
Meulman T. SOPS 2.3
Modoni A. SOPS 1.12, SOPS 2.12
Moll A. SOPS 2.5, SOPS 2.13
Mungai F. SOPS 1.14
Murmura B. SOPS 2.6
Mylanus E. SOPS 2.3

N
Nadtochiy A. SOPS 1.16, SOPS 2.9
Namdar Pekiner F. SOPS 3.1
Nardi C. SOPS 1.14
Nicolino G. SOPS 1.15

O
Orefice I. SOPS 2.6
Orhan K. SOPS 2.11
Orhan Söylemez U. P. SOPS 2.4

P
Pai I. SOPS 2.2
Palumbo G. SOPS 2.10
Parlak S. SOPS 2.7
Pasqualoni R. SOPS 1.1
Patella F. SOPS 1.15
Pellini R. SOPS 1.1

Petrillo M. SOPS 1.15
Pietragalla M. SOPS 1.14
Poon D. M. SOPS 1.3
Pulickal G. G. SOPS 1.13
Purpura P. SOPS 3.5

**R**
Rammelsberg P. SOPS 3.7
Ravanelli M. SOPS 2.10
Restivo V. SOPS 1.11
Ricciardi C. SOPS 1.9
Rinaldi G. SOPS 1.10, SOPS 1.11
Rodolico V. SOPS 1.10, SOPS 1.11
Romeo V. SOPS 1.9
Rotar H. SOPS 3.6
Ruberto E. SOPS 1.1
Rues S. SOPS 3.7
Ruiters S. SOPS 2.11

**S**
Sanguineti G. SOPS 1.1
Sansone M. SOPS 1.15
Schreiber A. SOPS 2.10
Schwindling S. SOPS 3.7
Shaheen E. SOPS 2.11
Shaikh F. SOPS 2.5
Singh D. SOPS 1.13
Sinha D. SOPS 1.7
Soliman S. SOPS 2.5
Staibano S. SOPS 1.9
Stanzione A. SOPS 1.9
Strojan P. SOPS 1.5
Suh C. H. SOPS 1.4
Šurlan Popovič K. SOPS 1.5

**T**
Talmaceanu D. SOPS 3.6
Taravella R. SOPS 1.10, SOPS 1.11
Tofanelli L. SOPS 1.15
Tomei M. SOPS 1.14
Trigona A. SOPS 2.6

**U**
Ugga L. SOPS 1.8
Ulay G. SOPS 3.1

**V**
Van Der Walk P. SOPS 2.13
Van Der Woude W. J. SOPS 2.3
Varela M. SOPS 1.2
Vart P. SOPS 2.3
Verbist B. SOPS 2.3
Verda M. SOPS 1.6
Verde F. SOPS 1.9
Vidiri A. SOPS 1.1
Vozgoment O. SOPS 1.16, SOPS 2.9

**W**
Warne R. SOPS 2.8
Weber D. SOPS 3.7
Willaert R. SOPS 2.11

**Z**
Zabret U. SOPS 1.5
